# Influence of Charge and Heat on the Mechanical Properties of Scaffolds from Ionic Complexation of Chitosan and Carboxymethyl Cellulose

**DOI:** 10.1021/acsbiomaterials.1c00534

**Published:** 2021-07-15

**Authors:** Andreja Dobaj Štiglic, Rupert Kargl, Marco Beaumont, Christine Strauss, Damjan Makuc, Dominik Egger, Janez Plavec, Orlando J. Rojas, Karin Stana Kleinschek, Tamilselvan Mohan

**Affiliations:** † Laboratory for Characterization and Processing of Polymers, Faculty of Mechanical Engineering, University of Maribor, Smetanova Ulica 17, 2000 Maribor, Slovenia; ‡ Institute of Automation, Faculty of Electrical Engineering and Computer Science, University of Maribor, Koroska cesta 46, 2000 Maribor, Slovenia; § Department of Bioproducts and Biosystems, School of Chemical Engineering, Aalto University, Vuorimiehentie 1, Espoo 00076, Finland; ∥ Department of Biotechnology, University of Natural Resources and Life Sciences, Muthgasse 18, 1190 Vienna, Austria; ⊥ Slovenian NMR Center, National Institute of Chemistry, Hajdrihova 19, 1001 Ljubljana, Slovenia; # EN→FIST Center of Excellence, Trg OF 13, SI-1000 Ljubljana, Slovenia; ∇ Faculty of Chemistry and Chemical Technology, University of Ljubljana, Večna pot 113, 1000 Ljubljana, Slovenia; ○ Departments of Chemical and Biological Engineering, Chemistry, and Wood Science, Bioproducts Institute, University of British Columbia, 2360 East Mall, Vancouver, British Columbia V6T 1Z4, Canada; ◆ Institute of Chemistry and Technology of Biobased System (IBioSys), Graz University of Technology, Stremayrgasse 9, 8010 Graz, Austria

**Keywords:** porous scaffolds, chitosan, carboxymethyl cellulose, charge complexation, polyelectrolytes, freeze-drying, dehydrothermal treatment, mesenchymal stem cells, tissue engineering

## Abstract

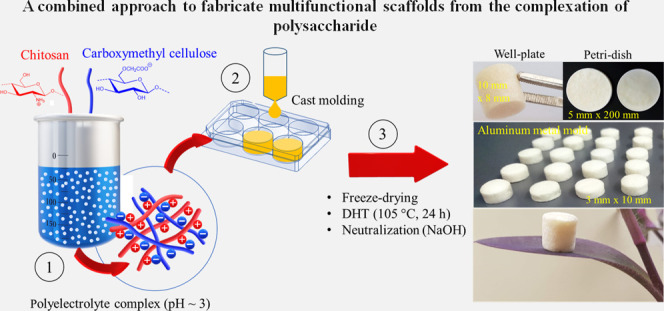

As one of the most abundant, multifunctional biological polymers, polysaccharides are considered promising materials to prepare tissue engineering scaffolds. When properly designed, wetted porous scaffolds can have biomechanics similar to living tissue and provide suitable fluid transport, both of which are key features for in vitro and in vivo tissue growth. They can further mimic the components and function of glycosaminoglycans found in the extracellular matrix of tissues. In this study, we investigate scaffolds formed by charge complexation between anionic carboxymethyl cellulose and cationic protonated chitosan under well-controlled conditions. Freeze-drying and dehydrothermal heat treatment were then used to obtain porous materials with exceptional, unprecendent mechanical properties and dimensional long-term stability in cell growth media. We investigated how complexation conditions, charge ratio, and heat treatment significantly influence the resulting fluid uptake and biomechanics. Surprisingly, materials with high compressive strength, high elastic modulus, and significant shape recovery are obtained under certain conditions. We address this mostly to a balanced charge ratio and the formation of covalent amide bonds between the polymers without the use of additional cross-linkers. The scaffolds promoted clustered cell adhesion and showed no cytotoxic effects as assessed by cell viability assay and live/dead staining with human adipose tissue-derived mesenchymal stem cells. We suggest that similar scaffolds or biomaterials comprising other polysaccharides have a large potential for cartilage tissue engineering and that elucidating the reason for the observed peculiar biomechanics can stimulate further research.

## 1 Introduction

Three-dimensional (3D) scaffolds are considered as biomaterials for tissue engineering to support the biological functions of damaged tissues and organs.^1^ Current advances in materials science offer new opportunities for the development of novel scaffolds for the regeneration of various tissues.^2,3^ However, some challenges still remain and currently available artificial scaffolds do not perfectly mimic the native extracellular matrices (ECMs) and the required support for biological functions.^4^ Scaffolds made of polysaccharides have been used for the regeneration of various tissues, including skin, cartilage, or bone.^5^ They have gained attention due to their hydrophilicity, biodegradability, swelling, and biocompatibility.^5^ These scaffolds must provide biocompatibility, porosity, chemical cues, and mechanical support to guide and attach cells.^6,7^ Several polysaccharide-based biomaterials, including nanocelluloses,^3^ hyaluronic acid,^6,8^ alginate,^9^ cellulose,^10^ carboxymethyl cellulose (CMC),^10,11^ and chitosan (CS)^7^ have been used to prepare such scaffolds.^7,10−17^ For example, a combination with collagen has been used as functional wound dressing to improve wound healing.^18^ Moreover, CS/CMC biocomposites with hydroxylapatite,^15,19−21^ silver,^14^ wollastonite,^17^ bioactive glass,^12^ calcium phosphate,^16^ and *Cissus quadrangularis* plant extract^22^ have been used in bone tissue engineering. Chrysin-loaded CS/CMC scaffolds were fabricated to promote proliferation and differentiation of mesenchymal stem cells (MSCs).^23^

While CS is acid-soluble and derived from chitin, CMC salts are partially biobased and water-soluble.^24^ Both polymers exhibit pH-dependent properties, e.g., solubility and charge density, and accessible functional groups, which make them attractive for further chemical modification^25,26^ and especially ionic cross-linking.^21,27^ Moreover, they can be easily processed from aqueous or acidic solutions.^7,28^ However, when mixed at a pH value where both polymers are charged and dissolved (e.g., pH 4), precipitation usually occurs, leading to weak polyelectrolyte interactions. Although CS is charged at pH 2.5, CMC can be almost fully protonated at this pH, resulting in an almost uncharged cellulose derivative. This may allow for better miscibility and formation of an interpenetrating polymer network.

**Figure 1:**
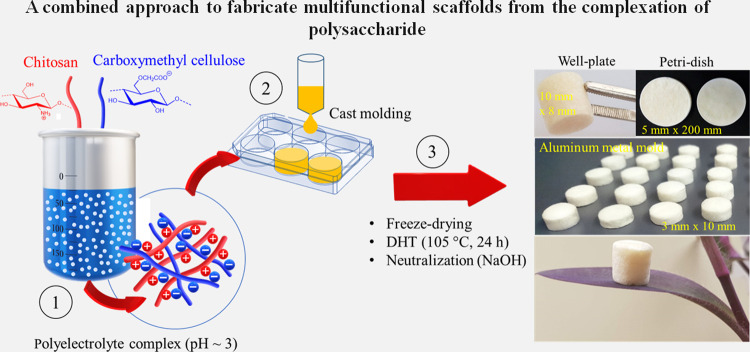
Illustration of the design leading to multifunctional and cross-linker-free biocomposite scaffolds by charge complexation of chitosan (CS) and carboxymethyl cellulose (CMC).

Scaffolds based on CMC and CS have been prepared via polyelectrolyte charge complexation (PEC) followed by freeze-drying^11^ and reported for tissue engineering applications.^13,29^ Even though they can easily be fabricated via PEC, most products lack dimensional stability or load-bearing capacity in biological environments under physiological conditions (37 °C, pH 7.4). Thus, the properties of scaffolds have been improved by chemical cross-linking, but this procedure often requires chemical modification or prior chemical treatment with reactive functional groups.^30−33^ This can be associated with cytotoxicity and require extensive purification. Therefore, an alternative process, namely, dehydrothermal (DHT) treatment in the dry state after PEC can be considered. DHT is solvent-free and frequently employed to improve the mechanical properties of biomaterials such as collagen.^34,35^ However, to date, no detailed studies have been reported on the influence of the charge ratio and subsequent heat treatment on the properties of scaffolds fabricated from the PEC of CS and CMC. Compared with other related works on the fabrication of CS/CMC scaffolds via PECs, in this work, we performed the charge complexation of CS and CMC at low pHs and at varying charge ratios (see Table 1) and solvent concentrations (acetic acid was used to dissolve both polymers). In addition, solvent- and chemical-free DHT treatment was employed to cross-link the functional groups of the polymers, which has not been previously reported in the literature for PESs CS/CMC scaffolds (see Figure 1). We hypothesize that the mechanism of charge complexation and the low pH at which complexation occurs as well as the heat treatment have a significant influence on the final mechanical properties, degradation, and swelling of the CS/CMC scaffold.

**Table 1:** Mass, Molar Charge Ratio, and pH of CS and CMC Solutions and Resulting Scaffolds

	CS			CMC			combined scaffold				
sample	*m* (g)	NH_2_ (mmol g^–1^)	pH	*m* (g)	COONa (mmol g^–1^)	pH	*m* (g)	CS/CMC (%/%)	NH_2_ + COONa (mmol g^–1^)	NH_2_/COONa (mol/mol)	pH
CS**100**	6	35.4	2.5	0	0	2.5	6	100/0	35.4	100/0	2.5
CS**60**	3.6	21.2	2.5	2.4	10.7	2.5	6	60/40	31.9	67/33	2.7
CS**50**	3	17.7	2.5	3	13.3	2.5	6	50/50	31.0	57/43	2.8
CS**40**	2.4	14.2	2.5	3.6	16.0	2.5	6	40/60	30.2	47/53	2.8
CS**0**	0	0	2.5	6	26.7	2.5	6	0/100	26.7	0/100	2.5

In this study, we investigate biocomposite scaffolds obtained by freeze-drying and DHT of CS/CMC after PEC. Different ratios of CS to CMCs were prepared, and charges, dissociation constants, chemical composition, and thermal properties of dry and hydrated scaffolds were analyzed in detail to elucidate the mechanism and pH of complexation. Time-dependent fluid uptake, degradation studies, and compression tests were performed using cell growth media under physiological conditions. The suitability of the scaffolds for tissue engineering was evaluated based on the viability and proliferation of human adipose tissue-derived mesenchymal stem cells (MSCs).

## 2 Experimental Section

### 2.1 Materials

Carboxymethyl cellulose (CMC, sodium salt, degree of substitution (DS)_COOH_ = 0.7, 90 kDa), chitosan (CS, 50–190 kDa), glacial acetic acid (AcOH), phosphate-buffered saline (PBS) (BioPerformance certified, pH 7.4), fluorescein isothiocyanate (FITC) isomer I, streptomycin, and penicillin were purchased from Sigma-Aldrich, Germany. Advanced Dulbecco’s modified Eagle’s medium (ADMEM) and fetal bovine serum (FBS) were purchased from Thermo Fisher, Germany. Ultrapure water (Milli-Q system, Millipore; *R* > 18.18 M Ω cm) was used for the preparation of all samples.

### 2.2 Preparation of Chitosan–Carboxymethyl Cellulose Scaffolds

Chitosan (CS) and carboxymethyl cellulose (CMC) were dissolved separately in 5–30% (w/v) acetic acid at given concentrations (see Table 1) so that both CS and CMC were mostly protonated, thus suppressing precipitation through charge complexation during mixing and allowing interpenetration of the two polymers. The solutions were stirred with a mechanical stirrer (150 rpm, IKA EUROSTAR 20) under ambient conditions until complete dissolution of the respective polymer. Afterward, each polymer solution was ultrasonicated for 15 min under ambient conditions to remove air bubbles. Then, the CMC solution was slowly added to the CS solution and stirred mechanically at 1500 rpm for 60 min. This polyelectrolyte complexation process led to a turbid colloidal dispersion that was then poured into polystyrene dishes (100 and 200 mm in diameter) and/or multiwell plates: (12- and 24-well: diameter = 10–15 mm, volume = 3.4 mL), or homemade aluminum templates (10 mm diameter and 3 mm height). They were then frozen at −25 °C for 48 h and lyophilized at 10^–3^ mbar and −25 °C for 48 h. The lyophilized scaffolds were designated according to the CS concentration ***x*** in the final scaffold (***x*** in CS***x***), from CS**0** to CS**100** (Table 1).

#### 2.2.1 Heating Treatment

The freeze-dried scaffolds were subjected to solvent-free dehydrothermal (DHT)-treatment by placing them in a glass container, covering them completely with aluminum foil, and storing them in a vacuum oven (Vacucell 22; MMM, Munich, Germany) for 24 h at 100 mbar in a temperature range of 40–120 °C to cross-link them.

#### 2.2.2 Neutralization

Heated and nonheated scaffolds (pH 2.5) were neutralized with sodium hydroxide (NaOH). Briefly, scaffolds were immersed in 200 mL of a 0.05–0.2 M NaOH solution for 30–60 min, and then in 200 mL of ultrapure water (pH 7.4) for 30 min under constant stirring. Rinsing with ultrapure water was repeated three times for each scaffold. The neutralized scaffolds were then stored in PBS (pH 7.4) for biological experiments (see Section 2.13). For all other experiments, the neutralized wet scaffolds were lyophilized further as mentioned above. In addition, the neutralized wet scaffolds were immersed in 10 mL of biofluid (ADMEM + 5% FBS + 100 IU mL^–1^ penicillin and 0.1 mg mL^–1^ streptomycin) containing phenolic red for 30 min at 37 °C with constant stirring. The color change of the biofluid was observed. Heated and non-heat-treated neutralized scaffolds were designated as “CS***x***/*N*”, and “CS***x***/*y*/*N*”, where ***x*** is the concentration of chitosan in wt %, *y* is the temperature of the DHT in °C and *N* indicates the applied neutralization. The freshly prepared scaffolds, with and without heat treatment, but non-neutralized are referred to as dry and non-neutralized scaffolds.

### 2.3 Scanning Electron Microscopy (SEM)

The morphology of lyophilized scaffolds was analyzed by field emission scanning electron microscopy (FESEM). Prior to imaging, all samples were pressed onto a double-sided carbon adhesive tape (SPI 116 Supplies). No sputtering was performed on the sample surfaces (nonconductive). A Carl Zeiss FE-SEM SUPRA 35 VP electron microscope was used. The images were recorded with an acceleration voltage of 1 kV at room temperature, which is sufficient to obtain SEM images with good resolution. The sample pore sizes (PS) were measured by analyzing the SEM images with the Image J1.47 software.^36^

### 2.4 Confocal Laser Scanning Microscopy (CLSM)

Biocomposite scaffolds (CS**50** and CS**40** before and after neutralization), were stained with an FITC solution (*c* = 10 μg mL^–1^, dissolved in ultrapure water, pH 7.4) and then analyzed in the hydrated (wet) state by CLSM. Thin slices of scaffolds were cut from selected areas (surface or cross section, relative to the position within the freezing chamber) and positioned on glass-bottom dishes (WillCo Wells, U.K.) mounted on a computer-controlled stage. They were positioned perpendicularly to the 209 (dry) objective of an inverted CFM Leica TCS SP5 II, equipped with the LAS AF software program. The samples were excited with an argon laser (kex = 490 nm), while the resulting signal was detected by two hybrid detectors (HyD), with a preset emission range of 500–550 nm. The image size was 512 × 512 pixels, and the images were scanned at a scanning speed of 290 frames s^–1^.

### 2.5 Porosity and Density of the Scaffolds

Porosity analysis was performed for DHT- and non-DHT-treated scaffolds before neutralization. The mass of the scaffold after drying was denoted as *M*_1_, and after ethanol absorption as *M*_2_. The volume of the scaffold was recorded as *V*_1_. To avoid measurement errors due to scaffold expansion, all scaffolds were placed in a container that limited their volume to excessive expansion.^37^ Assuming the density of ethanol as 0.789 g cm^–3^, the scaffold porosity was then determined according to eq 1
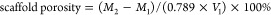
1The density of the scaffolds, ρ, was determined using the ratio of the weight *W* by sample volume^38^
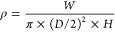
2where *D* is the diameter and *H* is the thickness of the sample.

### 2.6 Potentiometric Charge Titration

The potentiometric charge titration was performed with an automatic T70 two-burette titrator (Mettler Toledo) under an inert atmosphere (nitrogen gas bubble formation). The sample (1.5 mg mL^–1^) was titrated from acidic to alkaline between 2 < pH < 11 using 0.1 M KOH as titrant. The ionic strength of the analyte was adjusted to 0.1 M using KCl. All measurements were repeated three times. The amounts of charged groups present in the products were expressed in mmol g^–1^ sample. Determination of the amount of charged functional groups is described in detail elsewhere.^39^ Only a brief description is presented in this paper. In the titration system, as described above, the ionic species present are H^+^, OH^–^, their counter ions K^+^ and Cl^–^ as well as the species of interest, denoted as *A*_*k*_^*n*^, where *n* is the charge number and *k* is the enumerator. The total charge *Q*, due to the presence of *A*_*k*_^*n*^, is calculated using the electroneutrality condition according to eq 3
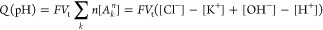
3where square brackets denote the ion concentrations in mol dm^–3^, *V_t_* is the total volume, and *F* is Faraday’s constant. The potassium- and chloride-ion concentrations, [K^+^] and [Cl^–^], respectively, are known from the titrant additions, while the hydrogen- and hydroxyl-ion concentrations, [H^+^] and [OH^–^], respectively, are measured with a pH meter. In a blank titration without the species of interest, only H^+^, OH^–^, K^+^, and Cl^–^ ions are present; thus, *Q* = 0 for any given pH. This allows replacing the [OH^–^] – [H^+^] term in eq 3 by the difference [K^+^]_blank_ – [Cl^–^]_blank_ and results in eq 4
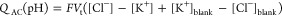
4The latter approach is recommended because it permits eliminating the error due to the presence of dissolved carbon dioxide in the titration system.

The titrant volume was normalized to the mass of the titrated samples and expressed as charges per mass (in mmol g^–1^) vs pH curve.

### 2.7 Attenuated Total Reflection-Fourier Transform Infrared (ATR-FTIR) Spectroscopy

The ATR-FTIR spectra of scaffolds were measured using a PerkinElmer FTIR System Spectrum GX Series-73565 at a wavenumber range of 4000–400 cm^–1^. A total of 32 scans were performed for all measurements with a resolution of 4 cm^–1^.

### 2.8 Solid-State Nuclear Magnetic Resonance (NMR)

Solid-state NMR spectra were acquired on an Agilent Technologies NMR System 600 MHz NMR spectrometer equipped with 3.2 mm NB dual resonance HX MAS probe. Larmor frequencies of the carbon nuclei were 150.75 MHz. ^13^C NMR chemical shifts were reported relative to tetramethylsilane (TMS) (δ 0.0 ppm). Samples were spun at 16 000 Hz.

### 2.9 Powder X-ray Diffraction (XRD)

The powder X-ray diffraction of polymers and scaffolds was investigated with an X-ray diffractometer (XRD, Bruker D8 Advance equipped with Cu Kα radiation). The scaffolds were cut into small pieces and deposited on the sample holder, and the XRD patterns were recorded at room temperature at scattering angle (2θ) = 4–70° with steps of 0.02° and a scan rate of 0.02° 2θ s^–1^.

### 2.10 Thermogravimetric Analysis (TGA)

The TGA was performed on a TGA 4000 thermal analyzer from PerkinElmer (Waltham, MA) instrument in a nitrogen atmosphere (20 mL min^–1^) of 40–900 °C at a heating rate of 10 °C min^–1^ using an Al_2_O_3_ crucible without a lid. The Pyris software, version 10.02.0468, was used for data evaluation.

### 2.11 Analysis of Swelling Capacity and Weight Loss

The swelling kinetics of the neutralized scaffolds (CS**50**/*N*, CS**50**/105 °C/*N*, CS**40**/*N*, and CS**40**/105 °C/*N*) in biofluid were investigated using a gravimetric method.^11,40^ The dried cylinder-shaped scaffolds (*d* = 10 mm, *h* = 12 mm) were weighed (initial weight, *W*_0_), immersed in 10 mL of biofluid (pH 7.4) at 37 °C. At predetermined time intervals (*W_t_*), the scaffolds were removed from the liquid, wiped dry carefully by a filter paper only on the surface, and weighed again. The swelling capacity at time *t* was calculated using eq 5.
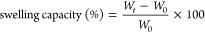
5To determine the weight loss upon contact with biofluid, the scaffolds (initial weight, *W*_0_) were placed in a beaker with 10 mL of biofluid at 37 °C and stirred at 200 rpm. At predetermined intervals, the scaffolds were removed from the biofluid, washed three times with ultrapure water, and lyophilized, as mentioned above. The remaining weight (RW) of the scaffolds was calculated as follows
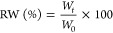
6where *W_t_* is the dry weight of the scaffold at a predetermined time.

### 2.12 Mechanical Strength Analysis

#### 2.12.1 Static Measurements

Unconfined compression tests were performed in wet and dry states. For wet state measurement, the scaffolds were previously equilibrated in biofluid for 2 h. The height and diameter of the samples were determined with a digital caliper gauge. Samples were measured in triplicate on a Universal Tester, Instron 4204 (Norwood), equipped with a static 1 kN load cell (Instron 2525 series) and 50 mm compression platens. The samples were compressed to 40% of their initial height at a rate of 2.4 mm min^–1^ and the elastic relaxation of the wet scaffolds was determined at a relaxation rate of 2.4 mm min^–1^. Data analysis was performed according to the literature:^11,41^ elastic modulus was determined from the initial slope of the stress–strain curve and the compressive strength equals the compressive stress of the samples at 30% compressive strain. Student *t*-test calculations for three independent means were used to determine the statistical significance in Figure 7D,E.

#### 2.12.2 Dynamic Measurements

Dynamic shear moduli of the wet samples were measured on a stress-controlled shear rheometer (Anton Paar MCR 302, Graz, Austria) using a 50 mm parallel plate geometry. Wet samples were prepared with a diameter of approximately 50 mm and a thickness of approximately 3 mm. Frequency sweep measurements (*n* = 3) were performed from 0.1 to 100 rad s^–1^ (30 measurement points in 30 s intervals) at a shear strain of 1%, with a variable gap size and a constant compressive force set to 0.9 N.

### 2.13 In Vitro Biocompatibility

The human mesenchymal stem cells (MSCs) used in this study were isolated from adipose tissue of a 65-year-old female donor after giving written consent, as described before.^42^ Isolation from human tissue was approved by the ethics committee of the Medical University Vienna, Austria (EK Nr. 957/2011, date: January 30, 2013). The biocompatibility of the scaffolds was evaluated using the 3(4,5-dimethylthiazolyl-2)-2,5-diphenyltetrazolium bromide (MTT) viability assay (Sigma-Aldrich, St. Louis, MO).^11,43^ All incubations were performed at 37 °C and 5% CO_2_ in a humidified incubator. The chosen scaffolds (CS**50**/105 °C/*N* and CS**40**/105 °C/*N*), with dimensions of 10 mm diameter and 3 mm height, were sterilized by UV light exposure (30 min each side), and then washed with PBS and minimum essential medium (MEM) α basal medium (Thermo Fisher Scientific, Waltham, MA) before use. MSCs at passage 5 were seeded at two different cell densities and cultivated under static condition in cell culture medium composed of MEM α, 2.5% human platelet lysate (PL BioScience, Aachen, Germany), 1 U mL^–1^ heparin (Ratiopharm, Ulm, Germany) and 0.5% gentamycin (Lonza, Basel, Switzerland).

For static cultivation, 100 μL of a 4 × 10^5^ cells mL^–1^ (low density) or 2 × 10^6^ cells mL^–1^ (high density) cell suspension were added on top of the scaffolds which were placed in the well of a 24-well plate and the scaffolds were incubated for 1 h to allow for cell attachment. After that, 2 mL of cell culture medium was added carefully to the respective wells. The cells were incubated for 5 days.

#### 2.13.1 Viability Assays

After that, the MTT viability assays were performed to assess possible cytotoxic effects of the scaffolds. For this, the scaffolds were rinsed with 37 °C PBS, transferred to a new plate, and covered with MTT solution (10% MTT and 90% MEM α) and incubated for 4 h on a horizontal shaker (200 rpm). Afterward, 10% sodium dodecyl sulfate (SDS) was added, the plate was incubated for 24 h, and absorption was measured with a plate reader (Tecan, Männedorf, Switzerland) at 570 and 630 nm. The absorption was corrected by subtracting the reference wavelength (570–630 nm). The values from scaffolds without cells were used as respective blank values and subtracted from the values of the seeded scaffolds.

#### 2.13.2 Live/Dead Cell Staining

The viability of cells was visualized with calcein-acetoxymethyl ester (AM) and propidium iodide (PI; both Sigma-Aldrich) staining. Briefly, samples were stained with calcein-AM (4 μM) and PI (8 μM). After washing with PBS, samples were investigated by fluorescence microscopy (Leica DM IL LED with Leica EL6000, both Leica Microsystems GmbH, Wetzlar, Germany).

#### 2.13.3 Statistical Analysis

Statistical analysis was performed using GraphPad Prism version 8.4.3 (GraphPad Software, La Jolla, CA). The data are represented as mean value ± standard deviation (SD). Student’s *t*-tests (nonparametric) with Dunnett test were carried out. The confidence interval was set to 95%, and significance was accepted at *p* ≤ 0.05.

## 3 Results and Discussion

### 3.1 Effect of Acetic Acid Concentration and Heat Treatment on Stability

We first focused on finding a suitable concentration of acetic acid (AcOH) to dissolve both polymers (CS and CMC) but to suppress most of the charges present in CMC to avoid immediate precipitation upon mixing both polyelectrolytes. We tested the concentration of AcOH, from 5 to 30% (w/v), but only 10% (w/v) AcOH resulted in the formation of uniformly sized scaffolds (see Figure S1), with no major holes or defects on either side of their surfaces. Such defect-free scaffolds are necessary for all types of physiochemical, mechanical, and biological evaluations. DHT treatment was expected to improve the dimensional stability, compressive strength, and elastic behavior of such scaffolds in complex physiological environments, and result in the formation of intermolecular cross-linking by condensation, either through the formation of amide or ester bonds.^11,34,35^ Compared to chemical cross-linking, DHT treatment is preferred because it does not involve solvents or toxic agents.^34^ Moreover, DHT treatment facilitates simultaneous sterilization at high temperatures and suitable exposure times.^34,35^ In our case, we assumed that the cross-linking reactions occurred between the hydroxyl and carboxyl or amino and carboxyl groups of the chitosan and CMC polymer chains (see Section 3.3). Photographs of the selected dry and non-neutralized scaffolds (CS**100**, CS**50**, CS**0**) after DHT at different temperatures (40–120 °C) for 24 h are shown in Figure S2. For scaffolds made of pure polysaccharides, no major color changes were observed, except at 120 °C. However, for CS**50**, a gradual color change (yellow to brown) was observed with increasing temperatures, which was more pronounced at 120 °C; the sample turned brown, which can be attributed to the formation of degradation products.^44^ The cross-linking temperature was varied from 40 to 120 °C, but the physicochemical and mechanical properties of the scaffolds were significantly improved only at 105 °C, and varying the time of the treatments enabled properties to be tuned (see Section 3.7). No major color change of the scaffolds was observed at 105 °C, hence, this temperature was used to prepare the scaffolds.

### 3.2 Scaffold Morphology and Porosity

#### 3.2.1 Dry Scaffolds

**Figure 2:**
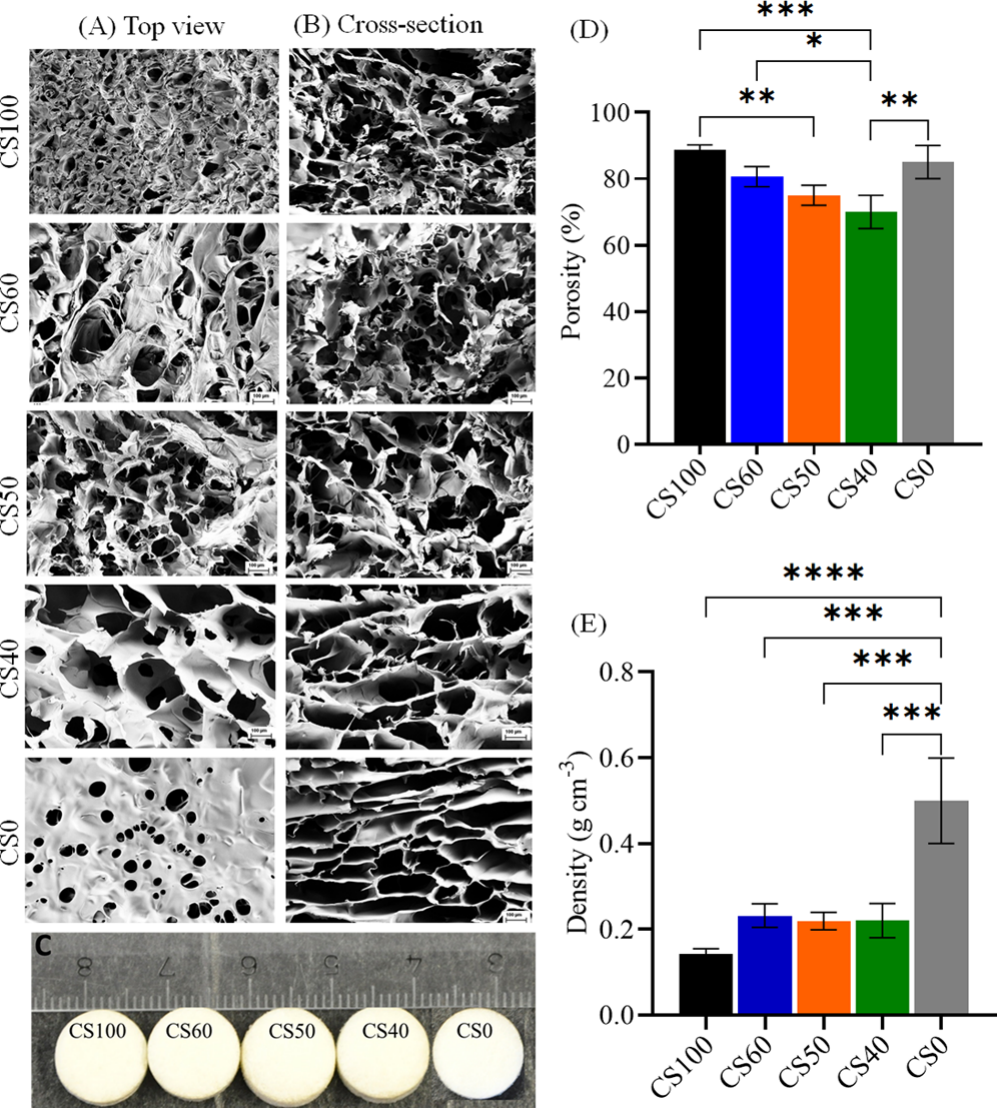
SEM images top (A) and cross section (B). (C) Photographs, (D) porosity, and (E) density of dry and non-neutralized scaffolds of chitosan (CS**100**), carboxymethyl cellulose (CS**0**), and chitosan–carboxymethyl cellulose biocomposites (CS**60**, CS**50**, and CS**40**). Statistically significant differences **p < 0.05, *p < 0.05.

The SEM images (A: top, B: cross section) and porosity (D) of the non-neutralized dry scaffolds (C, diameter: 10 mm, height: 3 mm) are shown in Figure 2. CS**0** showed more unidirectional pores (size: 100–300 μm) in cross section compared to the other samples (Figure 2B). It is suggested that the anionic nature and high solubility of CMC favored the slower and uniform nucleation of ice crystals during freeze-drying, and thus formation of unidirectional pores.^45^ The addition of CMC increased the porosity and pore sizes. Significant differences in morphology and porosity can be observed between the neat polymer, CS**50**, and CS**40**. The latter featured a more open-porous structure than the CS**50** (see Table 2). It is suggested that besides the electrostatic interaction, the CMC may interact differently with the chitosan at the interface at low pH (2.8), which could influence the ice templating during freeze-drying.^11,45^ This may lead to the formation of more open structures and morphology, especially in the case of CS**100** and CS**0**.

**Table 2:** Pore Sizes of the Non-Neutralized and Neutralized Scaffolds before and after DHT Treatment Obtained from SEM of CLSM Image Analysis

	SEM (dry samples)		CLSM (hydrated samples)	
sample	top (surface)	cross section	top (surface)	cross section
before neutralization				
				
CS**100**	50–100	100–300		
CS**60**	100–300	100–350		
CS**50**	80–250	100–350		
CS**40**	50–250	100–300		
CS**0**	50–300	100–320		
after heating and neutralization				
CS**50**/*N*	50–350	100–300	80–300	100–300
CS**40**/*N*	50–300	100–300	50–330	100–350
CS**50**/105 °C/*N*	80–350	50–300	50–350	40–300
CS**40**/105 °C/*N*	50–250	50–250	50–260	50–350

While the calculated average pore size ranged from 50 to 300 μm, all scaffolds had porosity in the range of 70–90% (D), which is suitable for most tissue engineering applications.^11,46^ The average pore sizes obtained in this work are comparable to the freeze-dried PEC scaffolds produced from CS/CMC (50–300 μm)^13^ or CS/CMC reinforced with bioactive glass (∼80 μm),^12^ silver nanoparticles (50–400 μm),^14^ hydroxyapatite nanoparticles (100–500 μm),^15,20,21^ calcium phosphate (35–290 μm),^16^ and wollastonite (∼100 μm).^17^ The density of dry chitosan (CS**100**) was 0.142 g cm^–3^ and increased to approximately 0.23 g cm^–3^ (Figure 2E, samples CS**50** and CS**40**) with CMC loading. These results can be compared with the values obtained for CS/hyaluronic acid scaffolds commonly used for both cartilage and bone tissue engineering applications.^47,48^

#### 3.2.2 Effect of Neutralization on Porosity

We aimed at neutralized acid-free and dimensionally stable scaffolds in a hydrated state (i.e., equilibrated in biofluid). Any excess or residual acid could cause undesired cytotoxic effects. All five (both heated and nonheated and DHT-treated) scaffolds, shown in Table 1 were therefore neutralized with NaOH at different concentrations and time intervals, as described in Section 2.2.2. The success of the neutralization was verified by storing the scaffolds in biofluid at physiological conditions. Several important findings were observed during the neutralization step: (i) only the bicomponent scaffolds CS**50** (containing 17.3 mmol NH_2_/13.3 mmol COOH) and CS**40** (containing 14.2 mmol NH_2_/16.0 mmol COOH) withstood the neutralization and retained their shape, whereas all other scaffolds (CS**100**, CS**60**, and CS**0**) collapsed after this treatment (see Figure S3) and could not be further used. We think that the strongest electrostatic interaction occurred due to an optimal amine/carboxyl ratio with balanced charges in CS**50** and CS**40**. (ii) Photographs of CS**50** immersed in cell growth media before and after neutralization are shown in Figure S4. While a yellow color (A) was observed for non-neutralized CS**50**, the completely neutralized (B) and acid-free CS**50**/*N* scaffold retained the initial pink color of the media. (iii) DHT-treated and neutralized scaffolds (e.g., CS**50**/105 °C/*N*) exhibited exceptional dimensional stability in sterile ethanol for more than 1 year (see Figure S5, Supporting information) without growth of mold/fungi compared to CS**40**/105 °C/*N*. NaOH concentrations and treatment times other than 0.1 M and 90 min resulted in ineffective neutralization or damaged the scaffolds. The focus of this work was set on CS**50** and CS**40**, and their properties were related to CS**0** and CS**100**.

**Figure 3:**
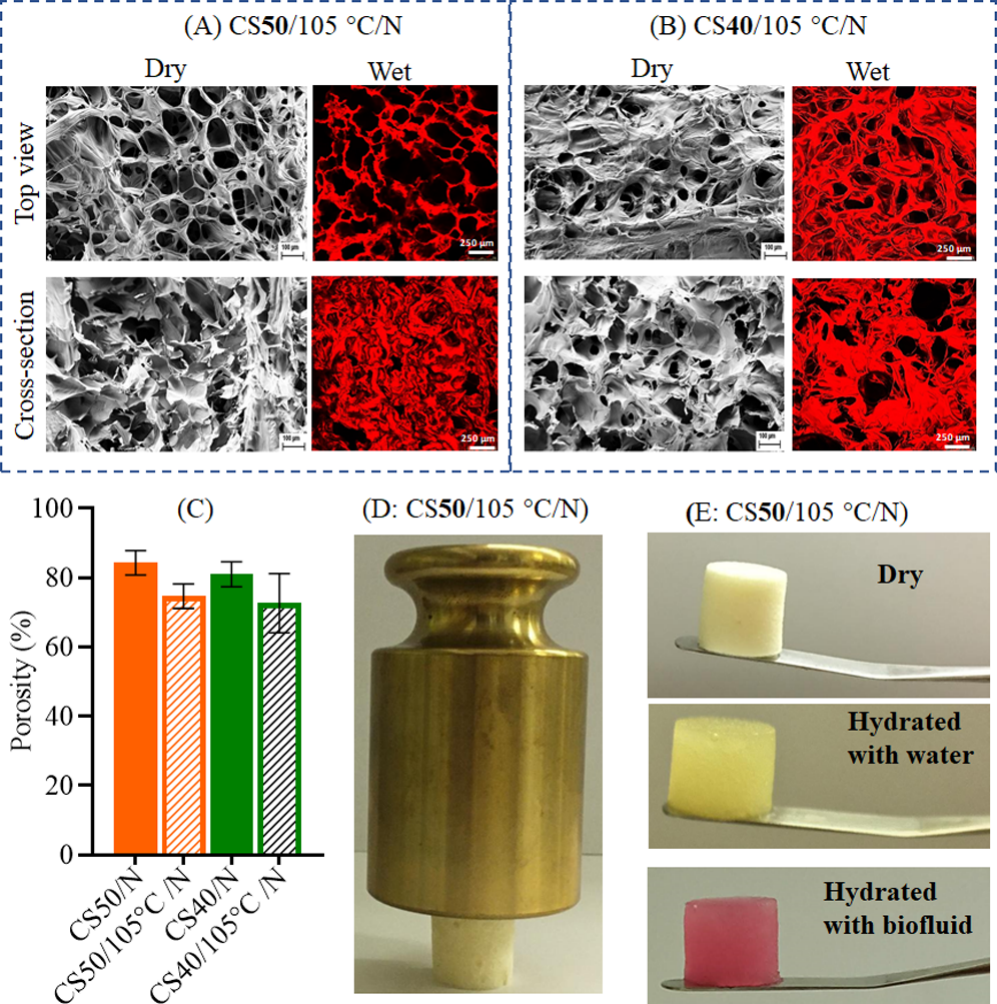
Top-view and cross-sectional images obtained by SEM (in dry state) and CLSM (in hydrated state) of heated and neutralized CS**50** (A) and CS**40** (B). (C) Porosity of heated and neutralized CS**50** (A) and CS**40** (B). (D) A scaffold with a density of 0.118 g cm^–3^ supported 500 g. (E) Photographs of dry CS**50**/105 °C/*N* scaffolds after hydration with ultrapure water and biofluid for 28 days.

Figure 3 shows the SEM and CLSM images of cross-linked (DHT-treated) and neutralized scaffolds (CS**50**/105 °C/*N* and CS**40**/105 °C/*N*) in the dry and wet states. For comparison, non-DHT-treated samples are shown in Figure S6. Even though no major differences in pore size were observed, heating and neutralization affected the morphology and strut shapes (see Figure 3). In the case of CS**50**/105 °C/*N*, the surface had a more open morphology and higher porosity compared to the bulk parts. This effect was less pronounced for CS**40**/105 °C/*N*. To visualize the morphological changes and porosity in the wet state, we performed CLSM of CS**50** and CS**40** (Figure S6) and after DHT (Figure 3). Before treatment, CS**50**/*N* and CS**40**/*N* showed a porous morphology in the hydrated state (Figure S6, CLSM), with interconnected fibrous networks, and a pore size (PS) ranging from 80 to 300 μm. These features were also observed for the DHT-treated scaffolds in the hydrated state (Figure 3, CLSM). The CS**40**/105 °C/*N* sample showed a slightly closed morphology and reduced pore sizes (50–260 μm), whereas CS**50**/105 °C/*N* (Figure 3, CLSM images) featured a more open structure with larger pore sizes (50–350 μm). The structure and pore size of the samples in the hydrated state was very similar to that in the dry state (SEM images, Figure 3). As mentioned in Section 3.2.1, the pore size of CS**50**/105 °C/*N* and CS**40**/105 °C/*N* in the dry and hydrated states can be compared with the pore sizes of most scaffolds obtained either from CS/CMC^13^ or from the latter incorporated with various reinforcing components.^14−17,20^ CS**50**/105 °C/*N* was physically resistant to deformation in the dry state as demonstrated by placing a 500 g weight on top with a density of 0.118 g cm^–3^. It retained its shape after hydration with water and biofluid at ambient conditions (Figure 3E).

### 3.3 Influence of Heat Treatment on Structure—NMR and IR Spectroscopies

**Figure 4:**
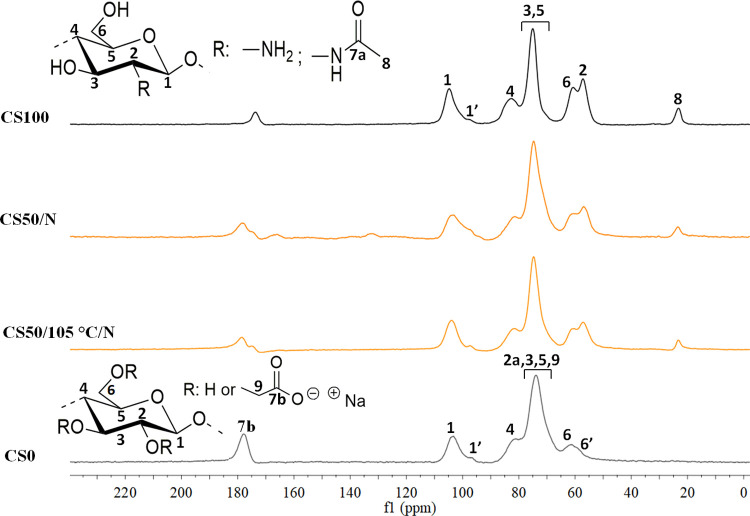
^13^C solid-state NMR spectra of chitosan (CS**100**), carboxymethyl cellulose (CS**0**), CS**50**/*N*, and CS**50**/105 °C/*N*.

To gain further insight into the chemical reactions of the functional groups of CS and CMC in the scaffolds upon DHT treatment,^34,35^ we performed solid-state NMR analyses (Figure 4) for CS**50**/*N* compared to the neat polymers. The ^13^C NMR spectrum showed characteristic signals at 174 ppm corresponding to the carbonyl (C=O) acetamide group for chitosan,^49^ and at 178 ppm attributed to the carbonyl carbon (C=O) of CMC.^11,50,51^ Although these signals were found in both CS**50**/*N* and CS**50**/105 °C/*N*, no new signals or significant shifts were detected. If cross-linking of functional groups has occurred, it is limited according to solid-state NMR. A similar phenomenon was observed for 3D-printed scaffolds prepared from the combination of nanobfrillated cellulose (NFC) and CMC.^11^ However, as we discussed below, significant changes in the mechanical properties and the amount of charges are visible, which could not be detected by solid-state NMR.

The ATR-FTIR spectra of the neat polymers and the dry and neutralized scaffolds of CS**50** and CS**40** before and after DHT are shown in Figure S7. Among other peaks, the neat CS (CS**100**) showed the characteristic absorption bands at 1653, 1378, and 1317 cm^–1^ corresponding to the C=O stretching of an amide bond (acetyl groups), C–H, and C–N stretching of the amide.^52^ For the neat CMC (CS**0**), the C=O stretching band of the carboxyl groups (COOH) was observed at 1580 cm^–1^, in addition to the other characteristic peaks. In the case of CS**50**/*N* or CS**40**/*N*, the C=O stretching band of the amide group was shifted to lower wavenumbers, 1640 cm^–1^, compared to CS**100**. We also observed the disappearance of the bands corresponding to the COOH groups of CMC (CS**0**), and the appearance of a common peak at 1550 cm^–1^ (N–H stretching vibrations), indicating the electrostatic interactions between the oppositely charged chitosan and CMC.^17^ After DHT treatment, a new band was visible at 1570 cm^–1^, which can be assigned to amide or ester bonds, formed by chemical condensation or induced formation of physical bonds based on electrostatic interactions.^53^

### 3.4 Influence of Heat Treatment on Charge

**Figure 5:**
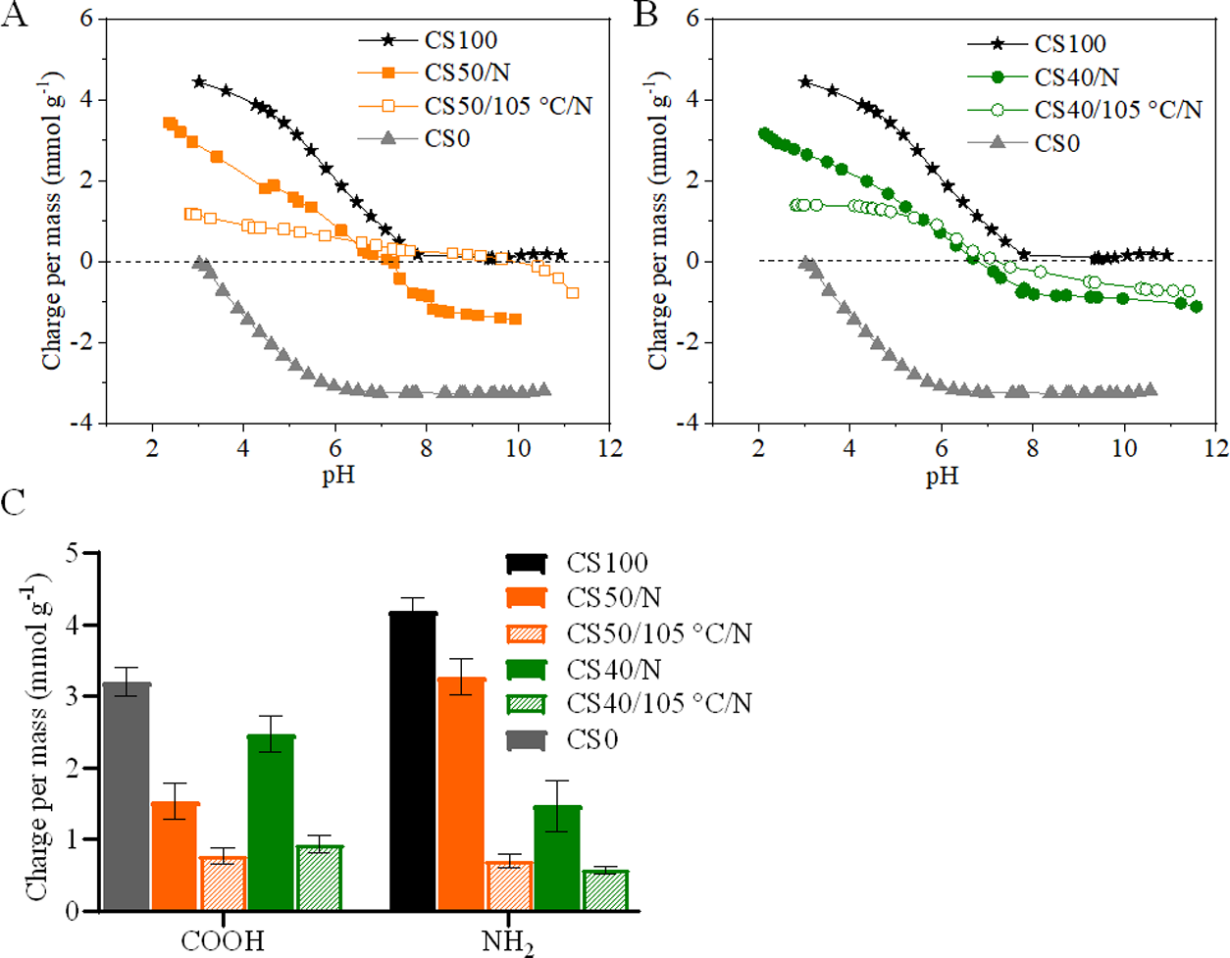
Potentiometric charge titration isotherms as a function of pH for CS**50** (A) and CS**40** (B) compared to CMC and CS. (C) Overall amino and carboxylate charge per mass for CS**50** and CS**40** and the effect of heat treatment.

To gain further insight into the influence of DHT, a pH-dependent potentiometric charge titration was performed. Figure 5A,B shows the charge/mass (*Q*/*m*, pH) isotherms. Only one slope was observed for CS**100** and CS**0** (Figure 5A,B), with p*K*_a_ values of 6.8 and 3.5 and total charges of 4.4 ± 0.4 and 3.2 ± 0.3 mmol g^–1^,^11,54,55^ respectively. On the contrary, CS**50**/*N* (A) and CS**40**/*N* (B) exhibited two slopes, which can be characterized by the difference in *Q*/*m* of two distinct plateaus. The changes in the slopes represent the protonation and deprotonation of the carboxylic and amino groups with a p*K*_a_ of 3.5 and 6.6, respectively, and total charges of 4.5 ± 0.3 and 3.2 ± 0.2 mmol g^–1^. The overall charges observed for neutralized and non-DHT-treated scaffolds were comparable to the theoretical amount calculated from neat CMC and CS (3.78 mmol g^–1^ for CS**50**, and 3.67 mmol g^–1^ for CS**40**). However, the DHT-treated scaffolds showed a 56–50% decrease in total charge (Figure 5C: CS**50**/105 °C/*N*: 1.22 ± 0.2 mmol g^–1^, CS**40**/105 °C/*N*: 2 ± 0.3 mmol g^–1^) without major changes in p*K*_a_ values. A similar result was obtained for the 3D-printed and freeze-dried scaffolds of NFC/CMC, which showed a 50% reduction in carboxyl charges after DHT treatment.^11^ DHT-treated and neutralized scaffold of CS**50** showed almost equal amounts of COOH (0.77 ± 0.1 mmol g^–1^) and NH_2_ (0.7 ± 0.1 mmol g^–1^) groups (see Figure 5C) compared to the DHT-treated CS**40** (COOH: 0.93 ± 0.1 mmol g^–1^, NH_2_: 0.57 ± 0.1 mmol g^–1^) (see Figure 5C). These results indicate that the DHT treatment led to a much stronger cross-linking of the functional groups of CS**100** and CS**0** in the CS**50**/105 °C/*N* scaffolds or that noncovalent cross-linking limits the number of accessible amino and carboxyl groups through titration. This kind of additive-free cross-linking can be of interest to obtain dimensionally stable scaffolds.

### 3.5 Influence of Heat Treatment on Crystallinity and Thermal Properties

The influence of the DHT treatment on the crystallinity of neutralized CS**50** and CS**40** was investigated by powder XRD measurements. Figure S8 shows the XRD diffractograms of the neat polymers (CS**100** and C**S0**), and the composite scaffolds, CS**50**/*N* and CS**50**/105 °C/*N*. Both composites differed from the neat diffractograms and the characteristics peaks for CMC and chitosan disappeared or broadened indicating a more amorphous structure of the composites and a homogeneous dispersion of the neat polymers in the composite matrix.^56,57^Figure S9 shows the results of the TGA and its derivative (dTG, change in mass loss rate) of the neat polymers (CS**100** and CS**0**) and the scaffolds between 40 and 900 °C before and after DHT treatment. The observed thermal behavior for the neat polymers, in three stages, is discussed in detail in the Supporting Information (Figure S9). In general, a significant variation in the degradation pathways and melting temperature was observed for the CS**50**/*N* and CS**40**/*N* scaffolds compared to the neat polymers. The peaks in dTG, seen between 100 and 200 °C for the neat CMC and chitosan, did not appear for the composite scaffolds, which is the result of CMC decarboxylation reactions^11,58^ and the onset decomposition of chitosan,^59^ among other factors. Otherwise, both neutralized scaffolds, before and after DHT treatment, showed a similar thermal decomposition pattern as the neat polymers. However, the total weight loss of all composite scaffolds was only 24 wt %, almost half that of the neat chitosan or CMC. We believe that the increased thermal stability is further proof for the occurrence of strong interfacial bonding between the oppositely charged polymers, as observed in the case of chitosan and carboxylated NFC.^60^

### 3.6 Influence of Heat Treatment on Swelling and In Vitro Degradation

#### 3.6.1 Biofluid Uptake

**Figure 6:**
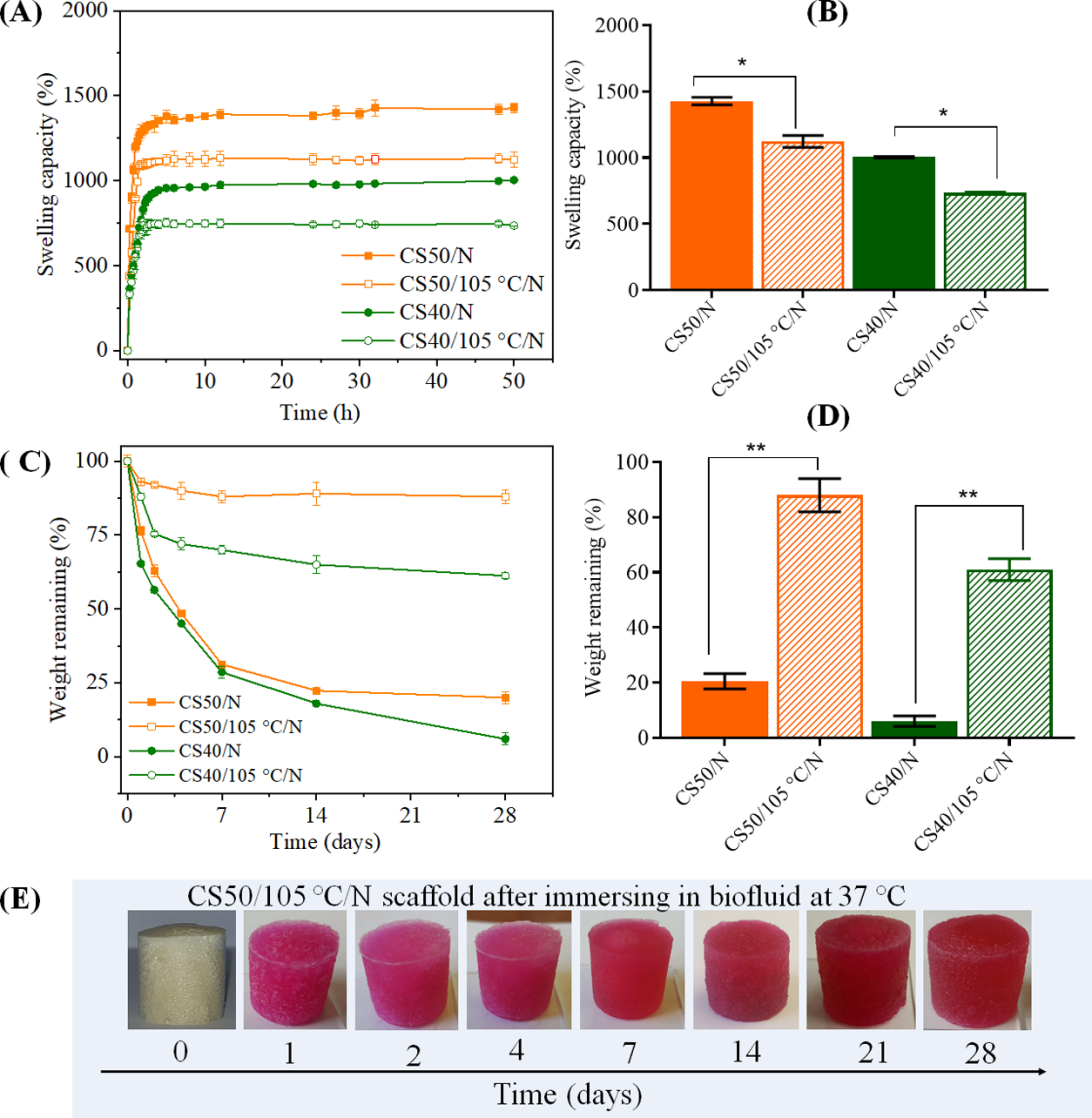
Swelling (A, B) and weight loss (C, D) of nonheated (CS**50** and CS**40**) and heated (CS**50**/105 °C/*N* and CS**40**/105 °C/*N*) upon equilibration in cell growth media at 37 °C. (E) Images of “CS**50**/105 °C/*N*” taken after the weight loss test at different times. Statistically significant differences **p < 0.05, *p < 0.05

The swelling kinetics of CS**50**/*N* and CS**40**/*N* before and after DHT are shown in Figure 6. The fluid uptake of all scaffolds increased rapidly in the first hours, followed by a steady state, which was reached more rapidly in the cross-linked scaffolds (Figure 6A). Maximum absorption was observed after 6 h, whereas it was reached in less than 2 h for the cross-linked scaffolds. Both non-cross-linked scaffolds showed higher swelling than the non-cross-linked samples (Figure 6A,B), whereas CS**50** had the highest uptake due to its higher amount of charged groups compared to CS**40** (see Figure 6C). The water absorption was in the following order: non-cross-linked (CS**50**: 1427 ± 28 g g^–1^ > CS**40**: 1003 ± 6 g g^–1^) and cross-linked (CS**50**: 1122 ± 44 g g^–1^ > CS**40**: 734 ± 6 g g^–1^). The lower (23–26 wt %) fluid uptake capacity of the DHT-treated scaffolds compared to the non-cross-linked ones further confirmed the occurrence of cross-linking reactions, which is well supported by the charge titration results (ca. 50–60% reduction in total charge was observed for the thermally cross-linked samples). The swelling capacity (>1000%) observed for non-cross-linked CS**40**/*N* and CS**50**/*N* scaffolds in good correlation with the values obtained for similar polysaccharide scaffolds, including NFC/CMC,^11^ CS/CMC scaffolds, those without^13,18^ and with reinforcing agents: silver nanoparticles,^14^ hydroxyapatite,^15^ etc. In addition to the high swelling and associated 13-fold mass increase, the CS**50**/105 °C/*N* scaffolds retained their structural and dimensional stability in the biofluid, even after 28 days (Figure 6E). This demonstrates excellent fluid-retention properties, limiting tissue fluid and nutrient loss and supporting tissue growth inside the scaffolds.

**Figure 7:**
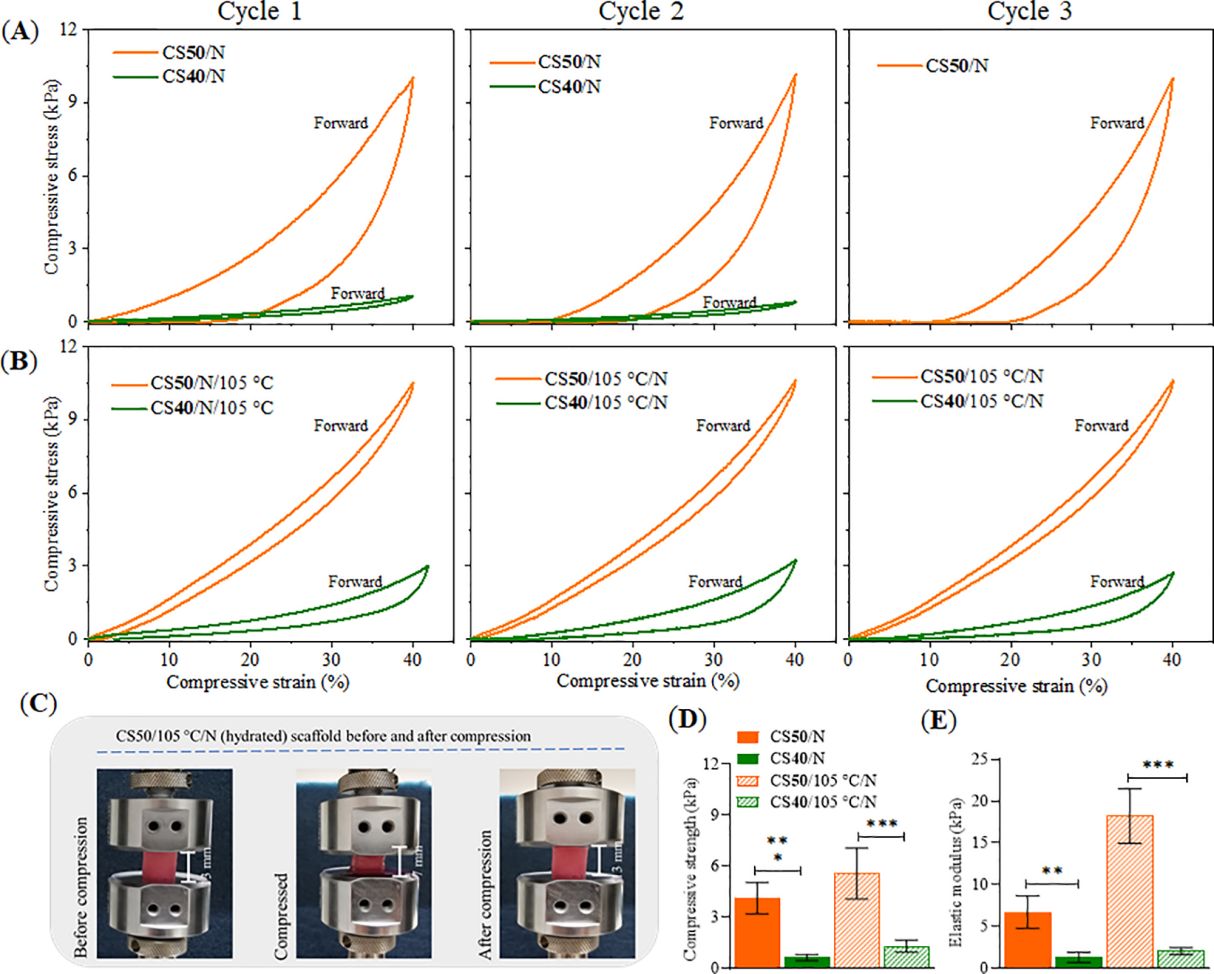
Stress–strain curves (A, B) and comparative mechanical properties (D, E) of neutralized scaffolds of CS–CMC composites and (C) photographs of the CS**50**/105 °C/*N* scaffold before and after compression test. Statistically significant differences **p < 0.03.

#### 3.6.2 In Vitro Degradation

Although both non-cross-linked scaffolds (CS**50** and CS**40**) showed similar degradation patterns, the CS**40** scaffold was more prone to degradation (Figure 6B). Interestingly, the degradation rate and pattern of the cross-linked scaffolds (CS**50**/105 °C/*N* and CS**40**/105 °C/*N*) were significantly different. For example, the weight losses of CS**50**/105 °C/*N* and CS**40**/105 °C/*N* were significantly lower (5- and 10-fold) than that of the non-cross-linked scaffolds after 28 days. CS**50**/105 °C/*N* featured the highest stability, with only 20 wt % loss after 28 days, compared to 40 wt % for CS**40**/105 °C/*N*. The total weight loss or degradation of our samples performed for 4 weeks in biofluid (containing a cocktail of various amino acids, vitamins, proteins, inorganic salts, glucose, etc.^61^), are almost comparable or even better than those of CS/CMC-based scaffolds reported in the literature where a degradation of 30–45 wt % was observed performed using water, PBS,^19,21^ simulated body fluid^20^ or in the presence of lysozyme^22^ at 37 °C. These results confirm that the cross-linking of chitosan and CMC chains by DHT treatment was successful and increased wet resilience, thereby preventing an uncontrolled and rapid degradation. Figure 6E compares the shape and dimensions of CS**50**/105 °C/*N* after different times in the biofluid. No significant change in shape or structural collapse was evident in the hydrated state over the entire period studied (0–28 days). Both the ionic complexation or interaction and the cross-linking of the polymer chains achieved by DHT treatment improved the dimensional and structural stability of the scaffold in biofluid under physiological conditions. Normally, such tremendous stability of scaffolds, especially in the biofluid, is achieved when additional chemical cross-linking agents were utilized, such as citric acid, genipin, acrylates, or carbodiimides.^62^ In contrast, we demonstrated that dimensional or structural collapse and disintegration of scaffolds in biofluid can be prevented by the combination of ionic complexation and by chemical cross-linking (amide or ester bonds) induced by DHT treatment at higher temperatures. Although we can state that this is the first study to confirm DHT-induced chemical cross-linking in the polysaccharide scaffolds, based on infrared and charge titration measurements, detailed investigations of these processes are currently underway, and a detailed discussion at this stage would be premature. Scaffolds with such versatile functional properties (e.g., stability, charges, etc.) obtained via a solvent-free process, have a high potential to be used in long-term in vitro, or even in vivo experiments.

### 3.7 Mechanical Properties

Figure S9 shows the mechanical properties of the dry scaffolds fabricated from the neat polymers (CS**100**: chitosan, CS**0**: CMC) and the composite scaffolds (CS**40** and CS**50**). The stress–strain curves typical of foam-like materials were observed for the neat scaffolds (CS**100** and CS**0**). The profiles started with a steep increase in compressive stress, i.e., elastic regime, followed by a plateau (plastic regime) at higher strains. CS**100** featured the highest compressive strengths (determined at 30% strain) of 265 ± 2 kPa, twice that of the neat CMC sample (CS**0**). The neat polymer samples exhibited high elastic response with compressive moduli in the range of 2–3 MPa. The composite scaffolds, CS**40** and CS**50**, showed a weaker elastic response with a lower slope in the initial strain values and featured elastic moduli of 0.5 and 1.2 MPa and compressive strengths of 116 and 180 kPa, respectively. These are significantly lower than those of their single components showing that the interpolymer bonding might be weaker than in the single materials (Figure S10). The results of neutralized dry scaffolds (DHT-treated) of CS**50** and CS**40** are shown in Figure S11. In general, both compressive strength and elastic modulus increased with increasing DHT time, and this was more pronounced at higher temperatures (100 and 120 °C) compared to lower temperatures (40–80 °C). This behavior was found for both scaffolds, but the CS**50**/*N* scaffold showed significantly increased mechanical properties at higher temperatures. These values are comparably high and exceed those reported for chitosan,^63,64^ chitosan-alginate,^65,66^ bacterial cellulose,^67^ ionically cross-linked poly(acrylic acid)/poly(allylamine hydrochloride),^68^ and chemically cross-linked CMC/collagen scaffolds.^69^ Despite the simple production process, the mechanical performance of the here-prepared biocomposites was in the range of high-strength scaffolds based on cellulose nanofibers^66,70,71^ and cellulose nanocrystals.^3^

Figure 7A shows the compressive strength of wet scaffolds CS**40** and CS**50** deformed repeatedly in three cycles. An exceptional difference can be observed as a function of the charge ratio between CMC and CS. CS**50** obviously leads to significantly stronger materials with higher compressive strength and elastic modulus (Figure 7D). In contrast, CS**40** disintegrates after the second compression cycle and could not be measured at the third compression. Moreover, a very significant increase in elastic moduli with increased charge balance is visible (Figure 7E), though full elastic recovery was not observed in the backward compression curve for both materials. The DHT treatment increased the absolute compressive strength, but more impressively, also led to a very significant increase in the elastic moduli of CS**50**, which can be explained by the presumed covalent cross-linking (Figure 7B,E) and a balanced charge ratio as determined from the charge titration results (see Figure 5C). This results in a very stable CS**50**/105 °C/*N* sample that can be repeatedly compressed in the wet state without disintegration (Figure 7C), and regaining of 100% of their original height when compressed to 40% normal strain. Interestingly, this unique spring-back behavior is comparable to the maximum physiological in vivo level before injury, as reported by other authors.^72^ This wet resilience was outstanding and demonstrated the inherent shape recovery properties of the scaffolds, and this resilience of the hydrated scaffold was likely due to the stronger effect of electrostatic charge repulsion between the charged groups at pH 7.4 in the composite and the associated hydrostatic (water) repulsion. This type of mechanism of water uptake during counterion influx occurs in cartilage tissue and explains the compression resilience of the latter tissue.^7,72^

We further compared the elastic behavior of CS**50** and CS**50**/105 °C further by rheological measurements (Figure S12). A similar observation can be made as in the unconfined compression tests; the cross-linking of CS**50**/105 °C led to a more pronounced elastic behavior, as indicated by the increased storage modulus from 10 to 22 kPa and the lower value of the loss tangent.

As shown in Table 3, the biocomposite CS/CMC scaffolds here-prepared had promising mechanical properties even in the hydrated state and were superior to hydrogels made from silk fibroin,^73^ neat chitosan,^63,64,74^ modified dextran,^75^ CMC/cellulose nanocrystals,^14^ and chitin nanofibers. They feature comparable properties to nanoparticle-reinforced hydrogels^70,71,73,76^ or other chemically cross-linked ones.^77,78^ Although the mechanical properties of scaffolds (especially when hydrated) are comparable to those of some other biomaterials, (as shown in Table 3), native cartilage (elastic modulus: 1–20 MPa)^79^ or bone meniscus matrix/horn (elastic modulus: 10–12 MPa)^80^ do have higher values in the wet state; but a match seems to be in reach with further modifications of the methods described here. Moreover, the mechanical properties of scaffolds could also be further increased by additional incorporation of a fibrous matrix, e.g., collagen,^34,35^ cellulose nanofibers,^10^ or cellulose nanocrystals,^10^ or by chemical cross-linking. These methods are currently being investigated in our labs.

**Table 3:**
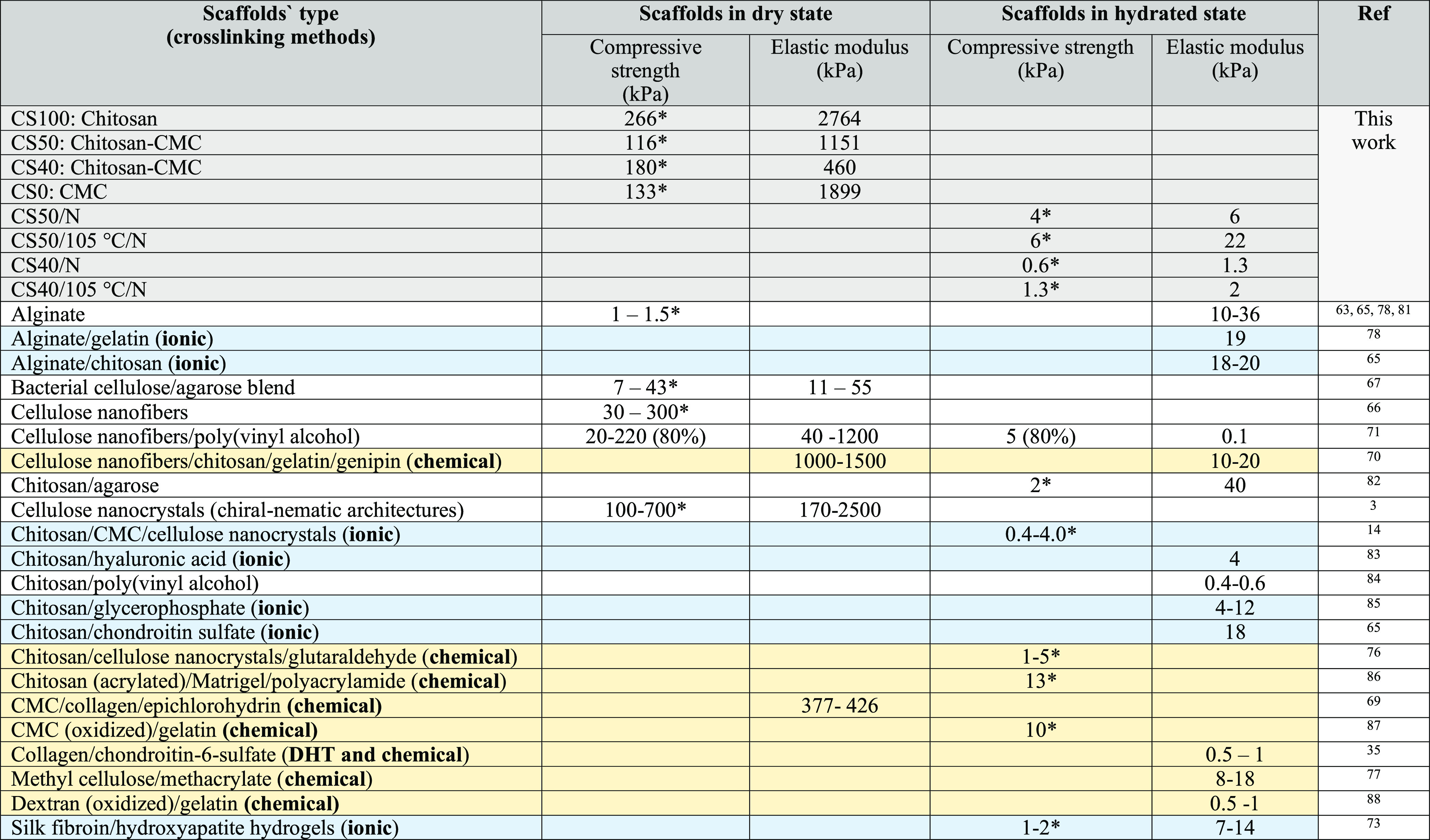
Comparison of the Mechanical Properties of Polysaccharide Scaffolds Obtained in This Study with Literature Values^81−88^^,^^a^

a *Strength values at 30% compressive strain. Specimens prepared in this work are highlighted in purple color, and literature values of ionically and chemically cross-linked are in blue and yellow colors, respectively.

### 3.8 Biocompatibility

**Figure 8:**
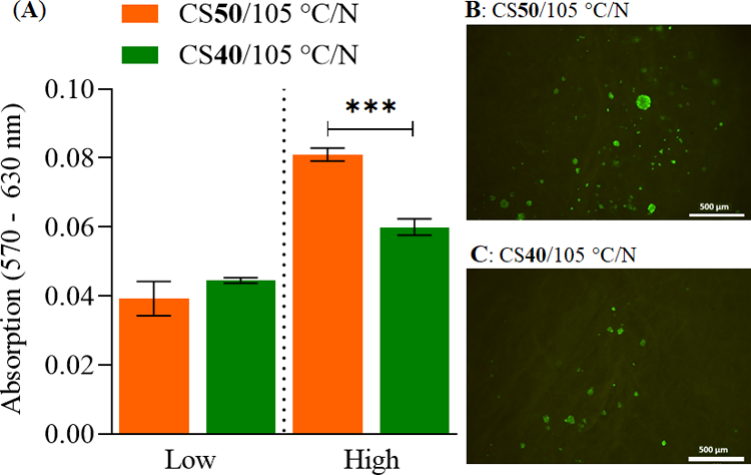
MTT viability assay of mesenchymal stem cells (MSCs) after 5 days of cultivation on CS**50**/105 °C/*N* and CS**40**/105 °C/*N* at low (40 000 cells per scaffold) and high (200 000 cells per scaffold) (A). Fluorescence images of a live/dead calcein-AM (green) and PI (red) staining of MSCs cultured on CS**50**/105 °C/*N* (B) and CS**40**/105 °C/*N* (C). Data are represented as mean ± SD (*n* = 4); * Indicates significant difference with a confidence interval of 95% and *p* ≤ 0.05.

To evaluate the biocompatibility of the scaffolds, we seeded MSCs at two different densities (low: 40 000 cells per scaffold, high: 200 000 cells per scaffold) onto CS**50**/105 °C/*N* and CS**40**/105 °C/*N* scaffolds. The incubation was performed under static condition for 5 days, as opposed to the standard exposure time of 24 h (according to ISO 10993-5), considering that in a real cell growth application, the cells would be in contact with the material surface for a longer period of time. As expected, the corrected absorption was higher in all samples with high cell density compared to low-density samples (Figure 8A). At a low density, no difference in viability was observed. However, the viability was significantly higher in CS**50**/105 °C/*N* vs CS**40**/105 °C/*N* at high cell density. It was assumed that cells in the “low” condition were too low in density, which resulted in a prolonged lag phase. In such case, the chosen time frame of 5 days was not enough to result in a significant difference in cell number.

Further, live/dead staining with calcein-AM (viable, green) and PI (dead, red) revealed that MSCs adhered to both types of scaffolds in the form of cell clusters, whereas slightly more cells were found on the surfaces of the CS**50**/105 °C/*N* scaffolds (Figure 8C). Since no dead cells were found on either scaffold, it can be stated that the porous scaffolds fabricated by our optimized procedure did not exhibit cytotoxic effects to the stem cells. This suggests that the chitosan–CMC biocomposite scaffolds are biocompatible, and might be used in the context in vitro cell culture models. Our results are in line with other studies where chitosan–CMC scaffolds were found biocompatible for human MSCs,^15^ MG63 (human osteosarcoma),^14^ and human dental pulp cells.^13^

## 4 Conclusions

We report on the influence of the charge ratio and heat treatment on the mechanical properties and stability of polysaccharide charge complexes. Light and mechanically strong, porous scaffolds were obtained from the ionic cross-linking of oppositely charged chitosan and carboxymethyl cellulose under acidic conditions. The low pH value during the complexation allowed for the formation of homogeneous interpenetrating polyelectrolyte complexes otherwise not accessible. Higher pH values, at which both polyelectrolytes are fully charged, would simply lead to coagulation, obviously avoiding the formation of cohesive polymer phases. Freeze-drying, neutralization, and dehydrothermal treatments without any additional cross-linkers were investigated and found to strongly increase stability for certain charge ratios. The most stable materials were fabricated at chitosan/carboxymethyl cellulose mass ratios of 40/60 and 50/50, which are close to equal charge ratios. They were highly porous, and their pore structure remained intact in the hydrated state. The stability strongly depended on the charge ratio, and a balance of this led to materials with very significant compressive strength, elasticity, and long-term stability in cell growth media and water. The scaffolds were compatible with human mesenchymal stem cells after 5 days of incubation and viability was higher when a balanced charge ratio was used. The fabrication methods and findings appear of value and could stimulate further investigations into the structure of charge complexes comprising polysaccharides. The exact mechanism leading to the exceptional stability and high elasticity are not fully understood, since an optimum seems to exist during which very stable complexes are formed. More detailed investigations in the complexation mechanisms and form, and especially in the influence of the polysaccharide molecular structure and the amount of charge are of interest. Combining other polysaccharides with a fibrous matrix could be a future research direction in this field, leading to potentially new applications of such materials not only for medical purposes.

## Supporting Information Available

Photographs of DHT-treated non-neutralized and neutralized scaffolds, photographs of CS**50**/105 °C/*N* immersed in sterile ethanol and biofluid, CLSM images of CS**50**/*N* and CS**40**/*N*, and XRD, TGA, and compression mechanical strength results of neat polymers and scaffolds (before and after neutralization and DHT treatment) (PDF)
