# Supplementary material for: Influence of Charge and Heat on the Mechanical Properties of Scaffolds from Ionic Complexation of Chitosan and Carboxymethyl Cellulose
Source: ACS Biomater Sci Eng. 2021 Jul 15;7(8):3618–32. doi: 10.1021/acsbiomaterials.1c00534 (PMC8396805; doi:10.1021/acsbiomaterials.1c00534)
Supplement: Supplementary file 1 — ab1c00534_si_001.pdf [file ab1c00534_si_001.pdf]

Supporting information

The influence of charge and heat on the mechanical properties of scaffolds from ionic complexation of chitosan-carboxymethyl cellulose

Andreja Dobaj Štiglic[†], Rupert Kargl[¥,‡,†,1], Marco Beaumont[$], Christine Strauss[ψ], Damjan Makuc[ζ], Dominik Egger[ψ], Janez Plavec[ζ,β,θ], Orlando J Rojas[$,∞], Karin Stana Kleinschek[¥,‡,1,*], Tamilselvan Mohan[¥,1,*]

[†]University of Maribor, Faculty of Mechanical Engineering, Laboratory for Characterization and Processing of Polymers, Smetanova Ulica 17, 2000 Maribor, Slovenia.

[‡]Institute of Automation, Faculty of Electrical Engineering and Computer Science, University of Maribor, Koroska cesta 46, 2000 Maribor, Slovenia.

[$]Aalto University, Department of Bioproducts and Biosystems, School of Chemical Engineering, Vuorimiehentie 1, Espoo 00076, Finland.

[ψ]University of Natural Resources and Life Sciences, Department of Biotechnology, Muthgasse 18, 1190 Vienna, Austria.

[ζ]Slovenian NMR Center, National Institute of Chemistry, Hajdrihova 19, 1001 Ljubljana, Slovenia.

[β]EN→FIST Center of Excellence, Trg OF 13, SI-1000 Ljubljana, Slovenia.

[θ]University of Ljubljana, Faculty of Chemistry and Chemical Technology, Večna pot 113, 1000 Ljubljana, Slovenia.

[∞]University of British Columbia, Bioproducts Instiute, Departments of Chemical and Biological Engineering, Chemistry and Wood Science, , 2360 East Mall, Vancouver, BC, V6T 1Z4 Canada.

[¥]Graz University of Technology, Institute of Chemistry and Technology of Biobased System (IBioSys), Stremayrgasse 9, 8010 Graz, Austria.

[1]Members of the European Polysaccharide Network of Excellence (EPNOE)

To whom correspondence should be addressed

***tamilselvan.mohan@tugraz.at, Tel: +43 316 873 - 32076.**

*karin.stanakleinschek@tugraz.at, Tel: +43 316 873 - 32070.

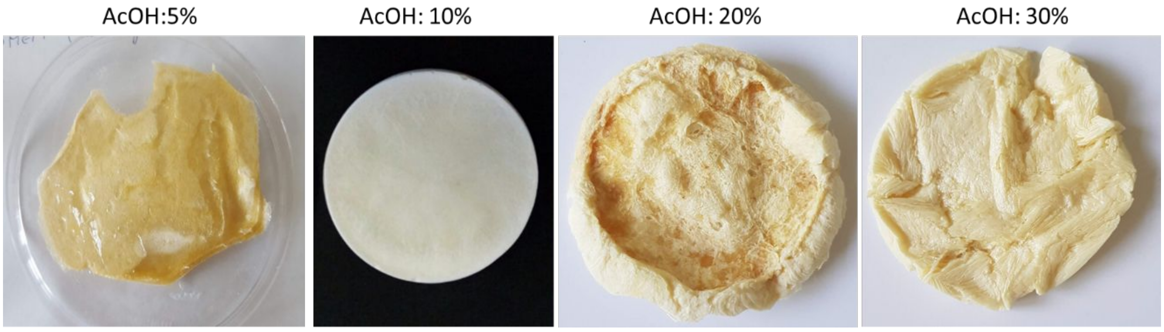

Figure S1. Photographs of CS0 scaffolds prepared using different concentrations of acetic acid.

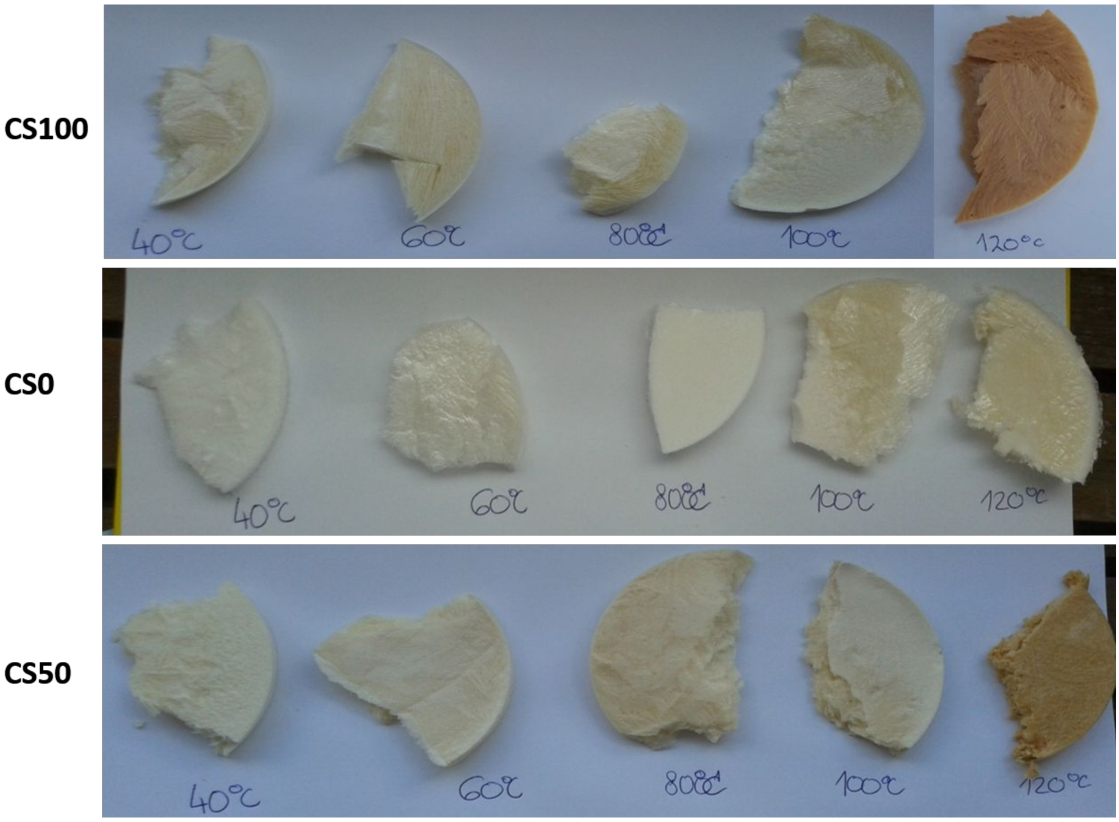

Figure S2. Photographs of CS100, CS50 and CS0 scaffolds after dehydrothermal treatment at various temperatures for 24 h.

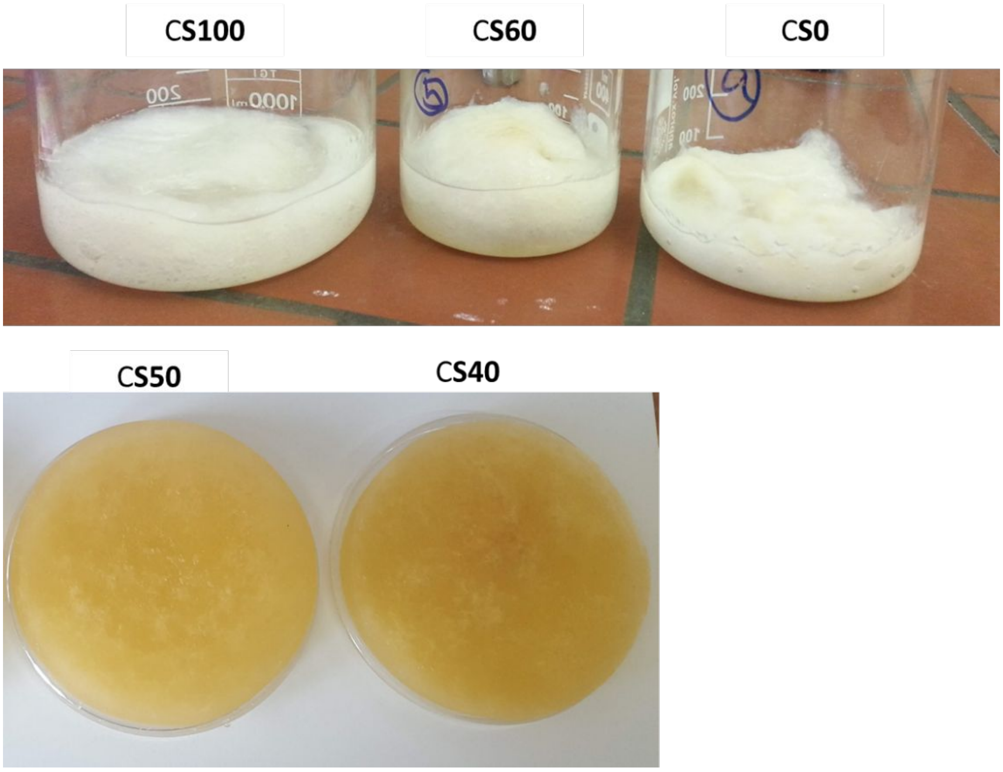

Figure S3. Photographs of neat and biocomposite scaffolds after neutralization with 0.1 M NaOH for 90 min.

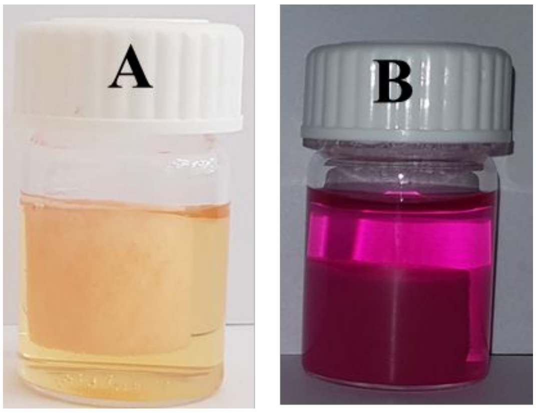

Figure S4. Photographs of CS50/105 °C (**A**) and CS50/105 °C/N (**B**) immersed in biofluid.

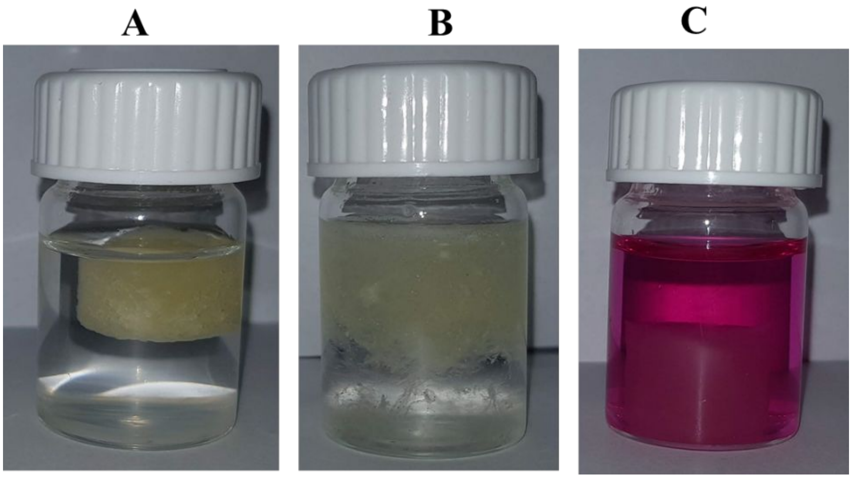

Figure S5. Photographs of DHT treated and neutralized scaffold of stored in sterile ethanol (**A:** CS**50**/105 °C/N, **B**: CS**40**/105 °C/N) for 1 year 6 months, and in biofluid (**C,** CS**50**/105 °C/N) for 4 weeks.

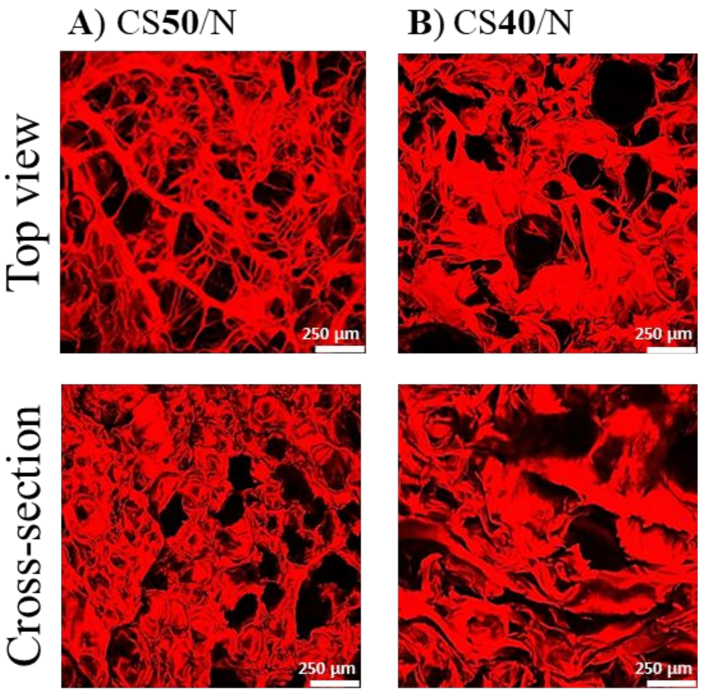

Figure S6. CLSM top view and cross-section images of non-heated and neutralized scaffolds of CS**50** (A) and CS**40** (B), measured in hydrated condition.

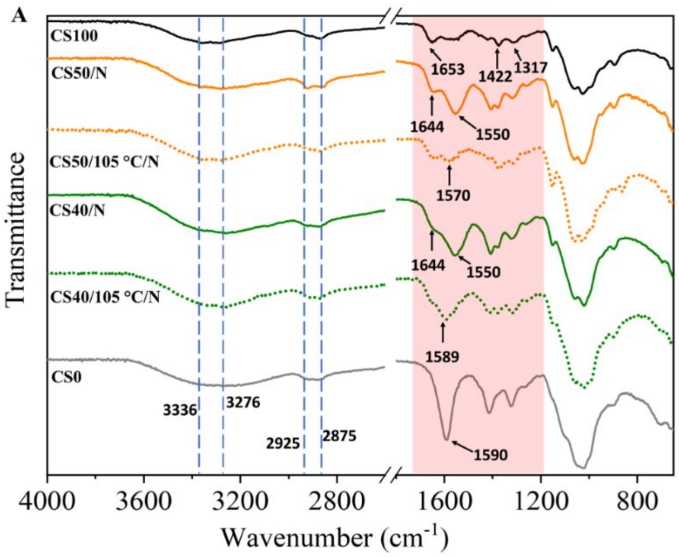

Figure S7. ATR-FTIR spectra of neat chitosan (CS100), carboxymethyl cellulose (CS0) and biocomposite scaffold, before (CS50/N) and after dehydrothermal treatment (CS50/105 °C/N).

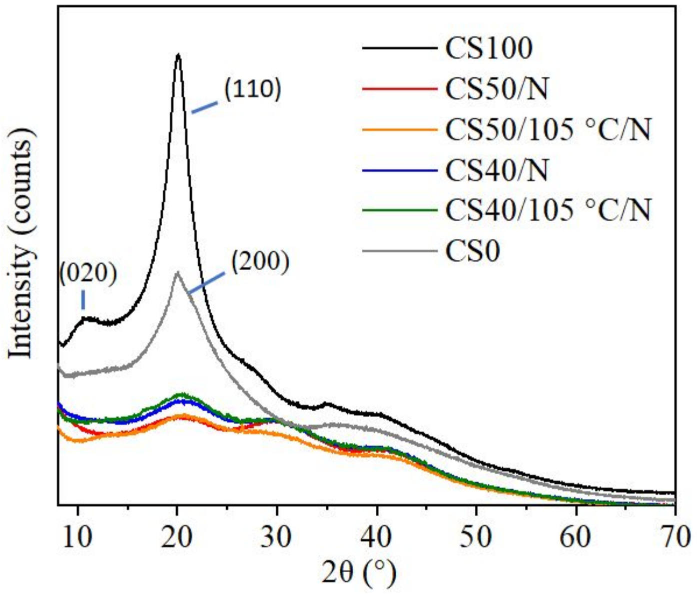

Figure S8. Powder XRD (D) spectra of polymers (CS100, CS0) and composite scaffolds.

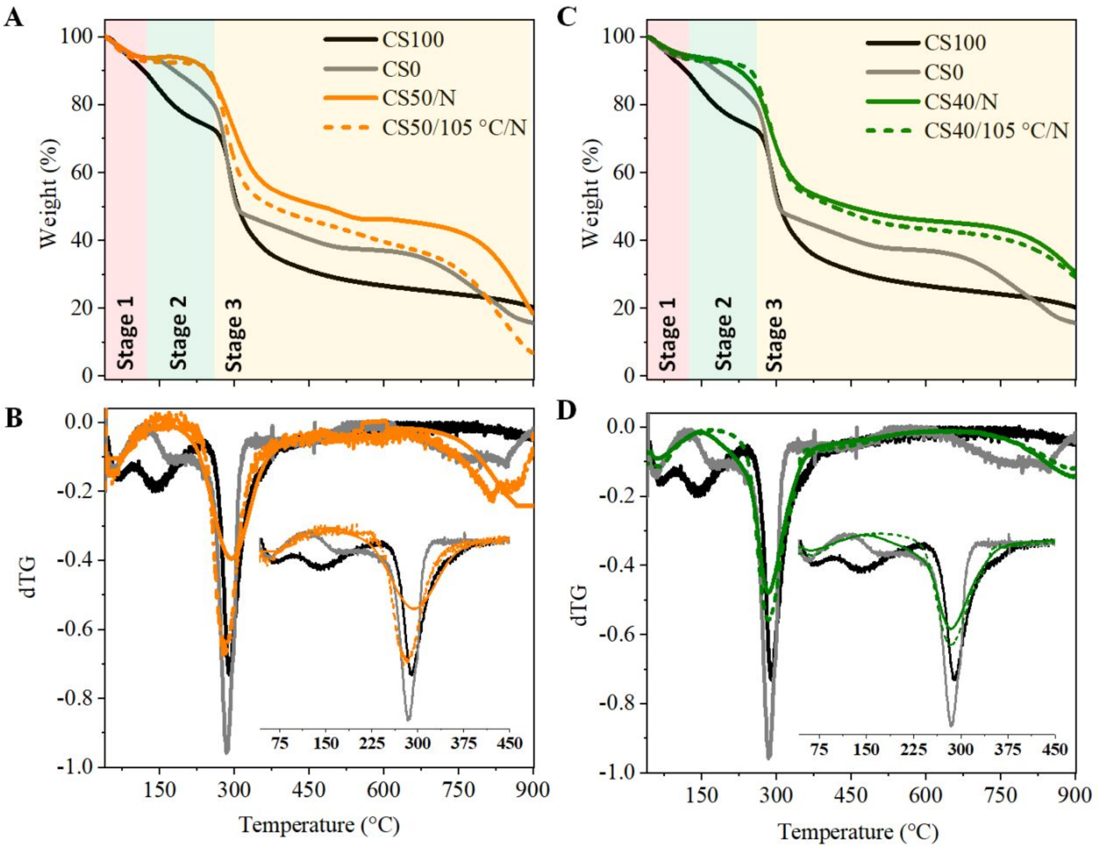

Figure S9. TGA and dTG curves of CS**50** (**A** and **B**) and CS**40** (**C** and **D**) neutralized scaffolds before and after DHT treatment. Results of neat polymers, chitosan (CS**100**) and carboxymethyl cellulose (CS**0**) are included for comparison.

The chitosan (CS100) degraded in thress stages (Figure 4A): The first stage occured between 40 and 120 °C and can be attricuted to removal of physically-bound H-bound water, corresponding to 3-5 wt% of the sample[1]. The second degradation stage was between 130-300 °C, with a loss of 16 wt% at 140 °C, was attributed to volatiles formed in chemical dehydration and depolymerization reactions of chitosan[2]. The third stage of decomposition (from above 300 °C) and a weight loss of 40% at 290 °C was attributed to the thermal decomposition of these intermediates. Similar to chitosan, three stages of degradation were also observed for the neat CMC (CS0). Between 50 °C and 110 °C (first stage), the dTG curve and thermogram showed a relatively large endothermic peak related to the glass-transition temperature of CMC and a

weight loss of less than 5% at 56 °C[3-4]. In the second stage between 120 and 200 °C, a weight loss of a 10% was observed at 178 °C, which was attributed to the removal of carbon dioxide from the polysaccharide chain and the degradation of the saccharide ring of CMC. The weight loss of 38% at 285 °C during the third stage (above 300 °C) was due to the breaking of C−O−C bonds in CMC.

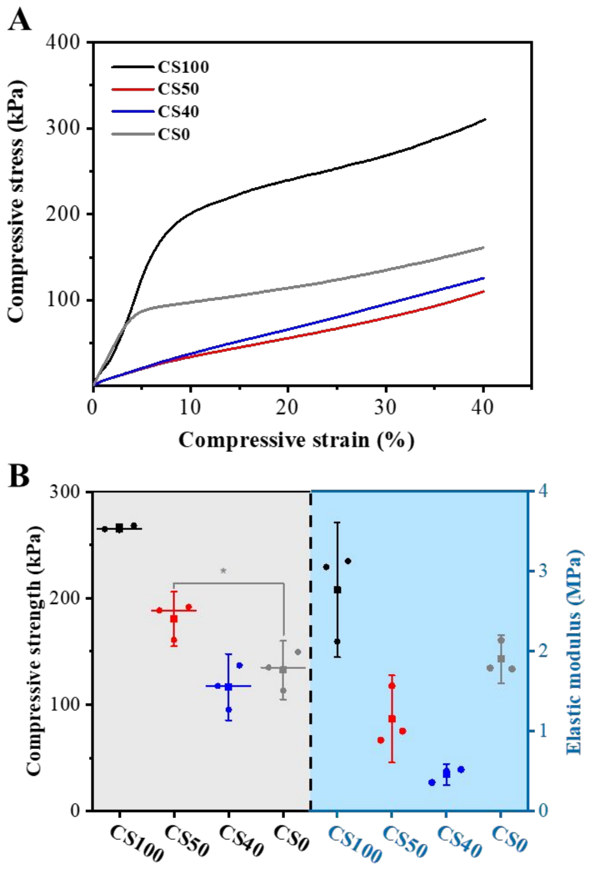

Figure S10. Compression stress and mechanical properties of non-neutralized scaffolds of CS (CS100), CMC (CS0) and CS-CS composites (CS50 and CS40). Statistically significant differences *$p < 0.02$ and **$p < 0.03$.

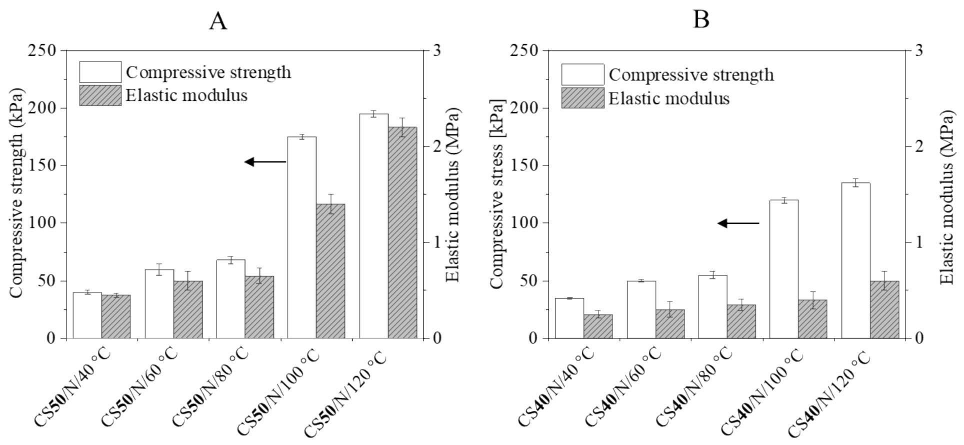

Figure S11. Mechanical properties of neutralized scaffolds of CS**50** and CS**40** heated at different temperatures.

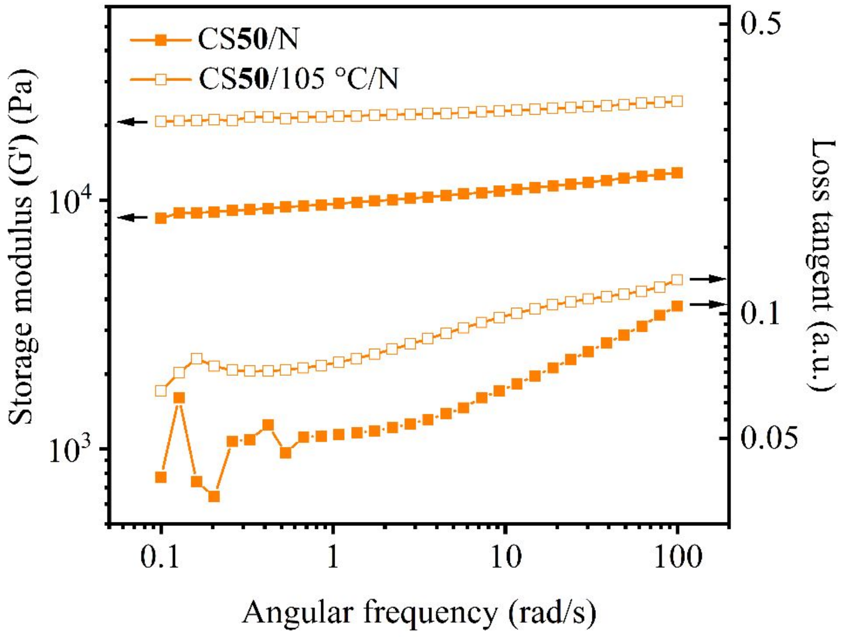

Figure S12. Dynamic rheology measurements of CS**50**/N and CS**50**/105 °C/N scaffolds.

References:

(1) Soni, B.; Hassan, E. B.; Schilling, M. W.; Mahmoud, B. Transparent bionanocomposite films based on chitosan and TEMPO-oxidized cellulose nanofibers with enhanced mechanical and barrier properties. *Carbohydrate Polymers* **2016,** *151*, 779-789, DOI: https://doi.org/10.1016/j.carbpol.2016.06.022.

(2) Pawlak, A.; Mucha, M. Thermogravimetric and FTIR studies of chitosan blends. *Thermochimica Acta* **2003,** *396* (1), 153-166, DOI: https://doi.org/10.1016/S0040-6031(02)00523-3.

(3) Basu, P.; Narendrakumar, U.; Arunachalam, R.; Devi, S.; Manjubala, I. Characterization and Evaluation of Carboxymethyl Cellulose-Based Films for Healing of Full-Thickness Wounds in Normal and Diabetic Rats. *ACS Omega* **2018,** *3* (10), 12622-12632, DOI: 10.1021/acsomega.8b02015.

(4) Mohan, T.; Dobaj Štiglic, A.; Beaumont, M.; Konnerth, J.; Gürer, F.; Makuc, D.; Maver, U.; Gradišnik, L.; Plavec, J.; Kargl, R.; Stana Kleinschek, K. Generic Method for Designing Self-Standing and Dual Porous 3D Bioscaffolds from Cellulosic Nanomaterials for Tissue Engineering Applications. *ACS Applied Bio Materials* **2020,** *3* (2), 1197-1209, DOI: 10.1021/acsabm.9b01099.
